# Photochemical Aging Induces Changes in the Effective Densities, Morphologies, and Optical Properties of Combustion Aerosol Particles

**DOI:** 10.1021/acs.est.2c04151

**Published:** 2023-03-21

**Authors:** Jani Leskinen, Anni Hartikainen, Sampsa Väätäinen, Mika Ihalainen, Aki Virkkula, Arunas Mesceriakovas, Petri Tiitta, Mirella Miettinen, Heikki Lamberg, Hendryk Czech, Pasi Yli-Pirilä, Jarkko Tissari, Gert Jakobi, Ralf Zimmermann, Olli Sippula

**Affiliations:** † Department of Environmental and Biological Sciences, University of Eastern Finland, Kuopio FI 70211, Finland; ‡ Joint Mass Spectrometry Centre, University of Rostock, 18059 Rostock, Germany and Cooperation Group Comprehensive Molecular Analytics, Helmholtz Zentrum München, München 81379, Germany; § Department of Chemistry, University of Eastern Finland, Joensuu 80101, Finland; ∥ Atmospheric Composition Research, Finnish Meteorological Institute, Helsinki FI-00560, Finland; ⊥ Finnish Meteorological Institute, Atmospheric Research Centre of Eastern Finland, P.O. Box 1627, Kuopio 70211, Finland

**Keywords:** combustion aerosol, soot, black carbon, residential combustion, morphology, photochemical aging, aerosol optics, wood, brown coal

## Abstract

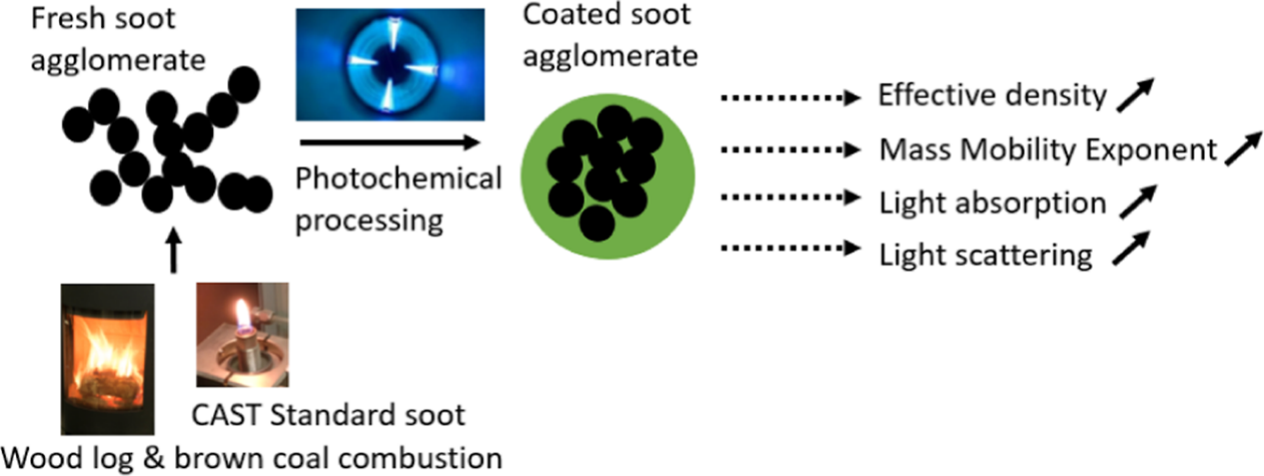

Effective density (ρ_eff_) is an important property describing particle transportation in the atmosphere and in the human respiratory tract. In this study, the particle size dependency of ρ_eff_ was determined for fresh and photochemically aged particles from residential combustion of wood logs and brown coal, as well as from an aerosol standard (CAST) burner. ρ_eff_ increased considerably due to photochemical aging, especially for soot agglomerates larger than 100 nm in mobility diameter. The increase depends on the presence of condensable vapors and agglomerate size and can be explained by collapsing of chain-like agglomerates and filling of their voids and formation of secondary coating. The measured and modeled particle optical properties suggest that while light absorption, scattering, and the single-scattering albedo of soot particle increase during photochemical processing, their radiative forcing remains positive until the amount of nonabsorbing coating exceeds approximately 90% of the particle mass.

## Abstract 2

Photochemical aging alters the effective densities and morphologies of residential combustion aerosol particles inducing changes in radiative forcing properties.

## Introduction

Many combustion processes emit high amounts of soot particles into the atmosphere. In general, soot particles are composed of elemental carbon chains of “primary particles”, which are often accompanied by condensed organic and inorganic material on their surfaces.^1^ Soot contains highly light absorbent black carbon (BC), which is one of the most notable anthropogenic short-lived climate forcers in the atmosphere.^2^ Emissions from solid fuel combustion in small, residential settings are a particularly important contributor to global air pollution and have substantial detrimental health impacts worldwide.^3,4^

Both climate- and health-related properties of combustion emissions are affected by particle morphology and chemical composition. Several morphological features of particles, such as surface area, fractal dimension, and mass mobility exponent, depend on the particle effective density (ρ_eff_). ρ_eff_ is an important parameter describing the transport properties of particles, including gravity and drag, and thus impacts the lung deposition of inhaled particles.^5^ Moreover, ρ_eff_ impacts light scattering- and absorption-related particle properties.^2,6,7^ Particle structure also impacts the ability of particles to take up water,^8,9^ which has further consequences considering the climate- and health-related properties of combustion emissions.^10,11^ Furthermore, particulate surface area is linked with the toxicological effects of ultrafine particles and is an important indicator of the potential adverse health effects of ambient air aerosols.^12^ Thus, changes in morphology influence particle deposition in the lungs and alter their direct radiative forcing efficiency (RFE) in the atmosphere.^13−15^

Once the combustion particles are released to the atmosphere, they are subjected to complex physical and chemical transformations. These processes, referred to as “atmospheric aging”, have been studied by measurements of ambient air^16,17^ and under more controlled conditions in laboratories using environmental chambers and oxidation flow reactors.^18−20^ During atmospheric aging, the gaseous organic species of the emission become oxidized and functionalized, leading to fragmentation or condensation of their reaction products and formation of particulate secondary organic aerosol (SOA).^21−23^ In addition, aging can cause multiform changes in the coating material, such as oxidation and functionalization, by heterogeneous reactions of the particulate phase.^20,24^ These changes alter particle behavior both in the atmosphere and in the respiratory system. For example, enhanced coating on soot particles is known to increase light absorption via the so-called lensing effect.^25^ Furthermore, atmospheric processes may lead to the formation or destruction of brown carbon (BrC), which is the organic particle fraction absorbing light at lower wavelengths.

Unit density (or some other constant) is often used in research as particle density over the whole particle size range due to simplicity or lack of knowledge. However, such an assumption is hardly ever true and can lead to discrepancies in data processing, for example, when particle number data are converted to mass size distributions^26^ or when the inhaled particle dose is estimated from particle number data.^5^ In practice, the ρ_eff_ of fresh soot decreases with increasing size due to the aggregated structure of the soot particles.^27−30^ In our previous study concerning fresh wood log combustion particles, ρ_eff_ was found to decrease with increasing size, while pellet combustion and burning of glowing wood embers produced particles with roughly constant density.^31^ Furthermore, the relationship between particle size and ρ_eff_ varies for ambient particles subjected to various levels of atmospheric processing.^32^ While fresh soot particles are highly aggregated, the formation of secondary aerosols during atmospheric aging has been noted to fill the voids of soot particles and form coatings on soot particles that compactify the agglomerate structure, thus increasing particle mass and ρ_eff_.^33−35^ Aggregated structures may also collapse due to evaporation of particle coatings in the atmosphere.^36,37^ Previous research on the photochemical transformation of soot morphology has been focused on laboratory-generated soot particles in the presence of specific precursors.^34,35,38−40^ However, only few studies exist considering real-world combustion aerosols.^41,42^ To the best of our knowledge, the effect of atmospheric aging on ρ_eff_ has not been studied previously for small-scale wood and coal combustion particles, although residential combustion is a major source of soot emissions worldwide.

The objective of this study is to determine how photochemical aging transforms ρ_eff_, morphology, and light absorption of residential combustion particles. For emission sources, we used a wood stove fired with (1) spruce logwood, (2) brown coal briquettes (BCBs), and (3) a combustion aerosol standard gas burner (CAST, Cast Jing Ltd., Switzerland^43^) as a well-known reference for soot particles. Photochemical transformation was simulated using the photochemical emission aging flow tube reactor (PEAR^44^). The effect of aging on ρ_eff_ and particle morphology is assessed by comparing the aged particles to the corresponding fresh particles. Finally, the effects of changing morphology on particle optical properties are estimated by both direct measurements of light wavelength-dependent absorption of the combustion aerosol and N-Mie core–shell modeling.

## Methods

### Particle Sources

#### Stove

A modern nonheat-retaining wood stove (Aduro 9.3, Denmark) was used for combustion of spruce wood logs (*Picea abies*) and BCBs manufactured from Lusatian coal (Rekord-Briketts G156; Lausitz Energie Bergbau AG, Germany).^45^ This type of stove is typically used for domestic heating. The nominal output of the stove was 6.0 kW. The use of the stove and fuels in these experiments is explained in detail elsewhere.^45^

#### CAST Burner

A combustion aerosol standard (CAST) burner (Cast Jing Ltd.)^43^ was used for soot particle production^46,47^ using propane gas as fuel. The air-to-fuel ratios (λ) of the CAST combustion were altered to vary the combustion conditions and to generate particles with varying properties. The operation of the CAST burner is described with more details in Supporting Information, S8.

### Instrumentation

#### Particle Measurements

The combustion aerosols were first diluted using a porous tube ejector sampling system by a factor of 29–55. Additional dilution by a factor of either 10 or 100 was carried out for aerosol measurements, by using ejector diluters, as described in Supporting Information Section S6, Figure S1. The measurements were carried out from both fresh aerosols and photochemically aged aerosols generated with the PEAR.^44^ Particle chemical compositions and coating factors (CFs)^48^ were derived by high-resolution soot particle time-of-flight aerosol mass spectrometry (SP-AMS, Aerodyne Research Inc.^49^). The SP-AMS was operated similar to Hartikainen et al. (2020)^50^ and described in Supporting Information, S7. In summary, the two vaporizer configurations were alternated every 120 s (including the 20 s particle time-of-flight mode). First, the nonrefractory submicron particles [NR-PM_1_, including organic aerosol (OA), nitrate, sulfate, ammonium, and chloride] were analyzed using the tungsten mode, where the thermal vaporizer was operated at 600 °C. Second, both NR-PM_1_ and refractory particles (namely, refractory black carbon, rBC) were studied using the dual vaporizer mode, with the combination of the thermal vaporizer and the continuous wave laser vaporizer (1064 nm^49^). CF was calculated as the ratio of the total NR-PM_1_ mass to the rBC mass. SP-AMS was only available in limited experiments; for others, a similar combustion period was used to estimate the CF. Collection efficiency of 1 was assumed. rBC was determined using high-resolution analysis of mass spectra to minimize interference by overlapping peaks. Elemental analysis of OA was performed using the improved-ambient method^51^ (Figure S2). The effect of photochemical aging on material densities of OA was approximated based on the O/C and H/C ratios following Kuwata et al. (2012).^52^ In addition, an electrical low-pressure impactor (ELPI, Dekati^53^) was used to measure the fresh particle number concentrations in the size range of 7 nm–10 μm in an aerodynamic dynamometer. The particle size distributions downstream of the PEAR were measured by an SMPS^54^ (DMA model 3080, CPC, TSI).

#### Density Measurements

The density measurement system and operation practices are described in detail by Leskinen et al. (2014).^31^ The definition of the ρ_eff_ used in this study and details of conducting the measurements are described in Supporting Information, Section S2. Briefly, ρ_eff_ of particles was determined using an APM (APM 3600, Kanomax Inc.)^30^ and an SPMS^54^ (equipped with CPC 3776 and DMA 3081, TSI Inc.) in series. The APM classifies particles according to their mass to charge ratio, (q m_p_^–1^), while the SMPS determines the number size distribution of the classified particles. ρ_eff_ is related to mass-mobility exponent (*D*_fm_) via the power law (eq 1), which illustrates the size dependency of the particle morphology.
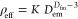
1where *D*_em_ = electrical mobility diameter and *K* = constant. Constant ρ_eff_ corresponds to spherical particles, which have a *D*_fm_ of 3.

A sample of diluted flue gas was directed to a stabilizing chamber with 60 dm^3^ volume to obtain a steady sample for the APM-SMPS system. The sample was guided through an aerosol neutralizer prior to the APM-SMPS system to achieve a known charge distribution for the particles. ρ_eff_ was calculated by comparing the measured mass of the particle to the virtual volume of a spherical particle with the same *D*_em_ as the measured particle. For spruce combustion, the APM-SMPS experiments were performed in the middle of the batch during the flaming combustion period. BCB experiments, however, can be characterized as either flaming combustion when filling of the sampling chamber was initiated 9 to 10 min after ignition of the fuel batch or residual char burning when BCBs were no longer burning with a visible flame but as glowing charcoal. Each stove measurement can be considered an individual experiment due to the fluctuating combustion conditions. A schematic of the experimental setup is available in Supporting Information, Figure S1.

### Transmission Electron Microscopy

Transmission electron microscopy (TEM, JEM-2100F; JEOL Inc., Tokyo, Japan) was used to study the morphology of the particles. TEM samples were collected from the stabilizing chamber using an aspiration sampler^55^ with a 0.3 Lpm flow rate. Particles were collected on holey carbon grids (S147-4 Holey carbon film 400 Mesh Cu; Agar Scientific Inc., USA).

#### Gas Analyzers

Carbon monoxide, carbon dioxide, nitrogen oxides, total organic gaseous compounds, a number of volatile organic compounds, and oxygen were measured directly from the flue gas of the wood stove using single gas analyzers and Fourier transform infrared spectroscopy (FTIR-DX4000, Gasmet) (Supporting Information, Section S5). Moreover, single-photon ionization time-of-flight mass spectrometry (SPI-ToF-MS^56^) was used to conduct untargeted analysis and semiquantification of aromatic volatile organic compounds (VOCs) in both stove and CAST burner experiments (Supporting Information, Section S1). The overall experimental conditions and gaseous emissions from the stove, including both FTIR and SPI-ToF-MS results as well as used solid fuels, are discussed in detail by Martens et al. (2021).^45^

#### Particle Optical Properties

Particle optical properties were directly measured by a seven-wavelength aethalometer (Aethalometer AE33, Magee Scientific^43^). Wavelength pairs 470 and 950 nm were used to derive the absorption Ångström Eeponent (AAE, eq 2,^57^), which describes the wavelength dependence of particulate light absorption (σ_a_).
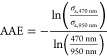
2

The AAE of pure black carbon is generally assumed to be ∼1, and AAE values greater than 1 indicate altered optical properties with enhanced absorption in the ultraviolet (UV) range due to coatings by nonabsorptive or weakly absorptive materials.^58,59^ The optically derived equivalent BC (eBC) concentration was determined from the absorption at 880 nm assuming a standard mass absorption coefficient (MAC) of 7.77 m^2^ g^–1^.

Wavelength-dependent particle absorption and scattering coefficients and asymmetry parameters were also calculated using the N-Mie core–shell model (Supporting Information, Section S3) for three different cases: fresh and aged brown coal combustion aerosol, which showed the highest increase in soot coating, and fresh CAST aerosol for which practically no coating was observed. Furthermore, the aggregate structure of the fresh aerosols was also considered by modeling the particle optical properties assuming that the aerosol consists of an external mixture of scattering organic and inorganic particles and absorbing BC particles by applying Rayleigh–Debye–Gans theory.^60,61^ Size distributions of soot and organic and inorganic aerosols were determined based on SP-AMS measurements (Table S2). For simplicity, we assumed that inorganics and organics form a mixed shell on the rBC core at each particle diameter measured with SP-AMS. Finally, the calculated absorption and scattering coefficients and asymmetry parameters were used to calculate the aerosol RFE (RFE = Δ*F* τ^–1^), i.e., aerosol forcing per unit optical depth.

#### Photochemical Emission Aging Flow Tube Reactor

The PEAR^44^ was used to simulate daytime atmospheric aging processes in two spruce, three brown coal, and three CAST experiments. The use of the PEAR and exposure conditions during APM-SMPS measurements are described in detail in Supporting Information, Section S4. A photon flux of 3 × 10^16^ photons cm^–2^ s^–1^ was estimated based on the UV lamp power and efficiency and the PEAR internal surface area (2.28 m^2^). Integrated OH exposures downstream of the PEAR were modeled based on the concentration of reactive gases in the exhaust.^62^ Median OH exposures during sampling were estimated to reach (0.9–2.6) × 10^11^ molecules cm^–3^ s, which correspond to 1–4 days of exposure at an ambient OH concentration of 10^6^ molecules cm^–3^.

## Results and Discussion

**Figure 1:**
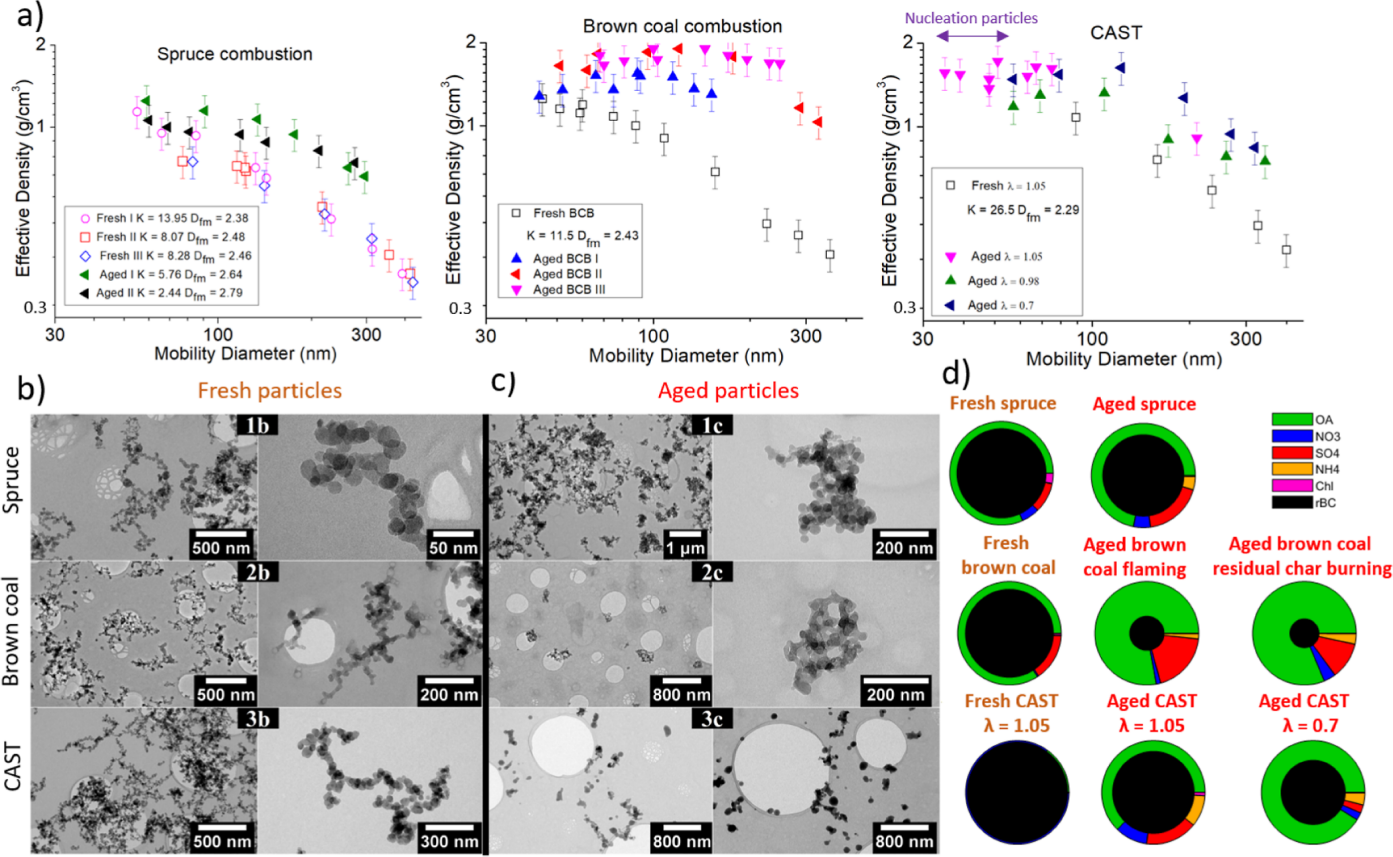
Morphology and chemical composition of the sampled combustion aerosol particles. (a) Effective densities of the studied aerosols as a function of mobility diameter (*K* and *D*_fm_ values are not presented when the effective density function does not follow the power law), example micrographs of (b) freshly emitted and (c) aged particles, and (d) diagrams of the particulate chemical composition measured with SP-AMS for sample cases.

In general, the combustion process in the stove can be considered typical for modern appliances. An overview of the studied emission properties is presented in Table 1. The combustion was relatively efficient, with an average modified combustion efficiency [CO_2_ (CO_2_ + CO)^−1^] ≥ 0.94 in all experiments. The fresh batch combustion emissions of logwood and BCBs contained 32.6–220 and 5.5 mg m^–3^ eBC, respectively. In addition, fresh BCB combustion exhaust included substantial amounts of sulfate (Figure 1) as a result of the high fuel sulfur content. The fresh CAST emissions were varied by changing the air availability. Independent of the chosen combustion conditions, the amounts of organics on the fresh CAST soot were low.

**Table 1:** Properties of the Emissions in Fresh Exhaust or in the Sampled Aerosol (with or without Photochemical Processing)^a^

	in fresh aerosol^d^					in the sampled aerosol (fresh/aged)^d^					
	sampling period (min^b^)	CO (mg m^–3^)	SO_2_ (mg m^–3^)	THC (mg m^–3^)	aromatic HC (mg m^–3^)	PN (cm^–3^)	OH exposure (molec. cm^–3^ s)	AAE	eBC (mg m^–3^)	CF	OA/rBC
instrument		Siemens	FTIR	FTIR	SPI-ToF-MS	ELPI		Ae	Ae	SP-AMS	SP-AMS
fresh spruce I	18–28 (f)	765	3.9	55	15.0	1.2 × 10^8^		1.22	38.2	0.31^c^	0.26^c^
fresh spruce II	14–19 (f)	1118	5.4	106	45.7	2.0 × 10^8^		1.27	220.7	0.24^c^	0.2^c^
fresh spruce III	38–45 (r)	1539	8.4	121	13.3	2.1 × 10^7^		1.24	32.6	0.27^c^	0.24^c^
aged spruce I	15–28 (f)	454	22.3	84	16.5	n/a	2.6 × 10^11^	1.22	415.2	0.65^c^	0.47^c^
aged spruce II	18–23(f)	188	6.6	32	7.0	n/a	3.4 × 10^11^	1.25	81.2	1.55^c^	1.31^c^
fresh BCB	9–21 (f)	5637	491	109	47.0	4.5 × 10^8^		1.75	5.5	0.46	0.36
aged BCB I	9–21 (f)	2899	358	55	75.3	1.3 × 10^8^	9.2 × 10^10^	1.41	2.9	20.9	14.5
aged BCB II	10–19 (f)	1208	366	48	9.2	2.0 × 10^8^	2.4 × 10^11^	1.49	4.2	12.6^c^	9.4^c^
aged BCB III	54–59 (r)	3526	113	65	121	2.7 × 10^8^	1.0 × 10^11^	1.46	5.7	12.2	9.9
fresh CAST^e^, λ = 1.05		n/a	n/a	n/a	4.9	n/a		0.94	14.1	0.05^c^	0.05^c^
fresh CAST^e^^,^^f^, λ = 0.7		n/a	n/a	n/a		n/a		1.85	3.6	0.09	0.09
aged CAST^e^, λ = 1.05		n/a	n/a	n/a	n/a	n/a	n/a	0.91	15.9	n/a	n/a
aged CAST^e^, λ = 0.98		n/a	n/a	n/a	3.4	n/a	n/a	0.95	3.6	0.59	0.37
aged CAST^e^ λ = 0.7		n/a	n/a	n/a	8.1	n/a	n/a	1.77	7.63	1.56^c^	1.42^c^

a Emission factors are presented as dilution-corrected concentration normalized to 13% excess O_2_ in flue gas. BCB is brown coal briquettes, THC is total hydrocarbons, PN is particle number concentration, HC is hydrocarbons, AAE is absorption Ångström exponent, eBC is equivalent black carbon, CF is coating factor, OA is organic aerosol, and rBC is refractory black carbon.

b Minutes from ignition of the latest batch.

c Determined from representative phases of combustion.

d Stove emissions are normalized to 13% flue gas oxygen.

e Emission data from the CAST burner are calculated using estimated dilution ratios.

f APM-SMPS measurement not performed on fresh low-lambda CAST soot. f = flaming and r = residual char burning period.

The density results are presented in Figure 1a as a function of particle size for fresh and aged emissions, while TEM micrographs (Figure 1b,c) illustrate the shape and size of the particles and SP-AMS results (1d) show the average composition of the particles. These results are discussed in detail in the following sections.

### Effective Density of Fresh Stove Emissions

Fresh wood and BCB combustion particles were clearly aggregated and exhibited mass-mobility exponents varying between 2.38 and 2.48 (Figure 1a). The size dependencies of ρ_eff_ for freshly emitted spruce and BCB combustion particles were roughly similar, with ρ_eff_ decreasing with increasing particle size in line with the power law (eq 1). The observed ρ_eff_s and *D*_fm_s were consistent with the ρ_eff_s and *D*_fm_s from fresh wood combustion aerosols determined in a previous study.^31^ Furthermore, they follow a roughly similar mass-mobility behavior as previously established for diesel soot.^30^ The qualitative analysis of PM morphology based on the TEM micrographs (Figure 1b) verifies that the fresh spruce combustion particles have a chain-like structure and consist mostly of soot primary particles, similar to our previous study.^31^ Additionally, the fresh BCB particles consisted mostly of refractory black carbon with a clear aggregate structure and primary particle sizes in the range of 10–20 nm. This finding is in agreement with Zhang et al. (2018),^63^ who also observed soot-dominated primary particulate matter from residential combustion of low-maturity brown coal.

Based on the elemental analysis of the OA, the composition and, consequently, the estimated bulk material density (approximately 1.3 g cm^–3^) of the fresh spruce combustion OA were similar to the bulk material density estimated previously for fresh spruce aerosol,^24,50^ while fresh BCB OA was more oxidized and thus slightly denser (approximately 1.4 g cm^–3^) compared to the spruce exhaust. The smallest measurable spruce and BCB combustion particles (approximately 50 and 40 nm in diameter, respectively) had ρ_eff_ values of 1.1 and 1.2 g cm^–3^, respectively, which are close to the estimated bulk material densities of the OA.

### Effective Density of Photochemically Aged Stove Emissions

The densities of the aged particles were roughly similar to the densities of the fresh particles in the smallest size ranges, where the particle size approached the estimated primary particle size. Primary particles obviously cannot collapse further, while the formation of a major coating that could impact ρ_eff_ would also shift the size toward larger ranges. For the larger particles, photochemical aging notably increased ρ_eff_, signifying prominent particle compaction. The observed increase in ρ_eff_ during photochemical processing can be explained by the formation of a coating on the soot particles resulting from functionalization and condensation of gaseous precursor compounds due to photochemical oxidation reactions.

For spruce combustion, the aged particles had larger mass mobility exponents and generally higher ρ_eff_ values compared to fresh emission, indicating that they were more closed in shape (Figure 1a). The ρ_eff_ of the spruce combustion particles, however, followed a power law even after aging. The average CFs of spruce combustion particles increased from 0.27 to 0.65. This enhancement can be attributed specifically to SOA formation since the ratio of OA to rBC increased similarly from 0.2–0.26 to 0.47–1.5. The changes in CFs were clearly dependent on the concentrations of freshly emitted soot and secondary aerosol precursors.

In contrast, photochemical processing of the BCB particles led to a relatively constant ρ_eff_ over the studied size range, which indicates spherical or somewhat compact particles. Their ρ_eff_ varied in the range of ∼1.2–2 g cm^–3^. This relatively large variation is due to the fluctuating batch combustion conditions. The CFs of aged BCB particles were above 12, which is notably higher than that for primary BCB particles or either fresh or aged spruce combustion particles. This thick coating was caused mainly by the high primary concentrations of gaseous aromatic species (Table 1) that lead to SOA formation and relatively low concentrations of soot in BCB combustion aerosols. In addition to SOA formation, the CFs of aged BCB particles were influenced by secondary sulfate formation (Figure 1c), which agrees with the high SO_2_ concentrations in the fresh exhaust (Table 1). During photochemical processing, SO_2_ forms sulfuric acid, which subsequently condenses onto primary particles. The high amount of condensed material in the aged BCB particles is also the likely explanation for the substantial increase in ρ_eff_. The condensing coating material can be expected to fill voids in the agglomerate structures, which may also lead to collapse of the agglomerate structure due to the increased surface tension.^64^

SOA is generally expected to be more oxidized than primary organic aerosol (POA), while POA also becomes more oxidized upon photochemical exposure,^20,24,50^ which would also increase the particle bulk material density. For spruce exhaust, photochemical aging increased the estimated OA material densities from 1.3 to 1.5–1.7 g cm^–3^. Spruce combustion particles, however, had a relatively thin coating after aging, which lessens the impact of the denser OA on the total particle density. For BCB, the estimated OA material density was similar for both aged and fresh aerosols (1.2–1.4 g cm^–3^), which is in agreement with the effective densities measured for aged BCB I, but lower than those for aged BCB II and III (Figure 1). Since OA formed 62–73% of the total chemically resolved particle mass in aged BCB particles, the results indicate that either the used equation underestimates OA material density for coal combustion OA or coal combustion PM contains some ash components, which increase the ρ_eff_. The TEM micrographs (Figure 1c) further support the notion that while spruce combustion particles remained agglomerated following photochemical exposure, the agglomerate structure of BCB particles collapsed upon aging. However, the electron microscopy grids are exposed to high vacuum and bombardment of electrons, causing some nonrefractory material to evaporate. As a result, the evaporation of the particle coating during microscoping can be observed as particles retaking an agglomerated structure (Supporting Information, Video S1).

### Effective Densities of Fresh and Aged CAST-Burner Soot

The CAST burner operated under standard operating conditions (λ = 1.05) produced fractal soot agglomerates with minor amounts of organic components in the fresh aerosol, which is in agreement with earlier studies on CAST soot.^43^ The CF (0.09) and organic coating (OA/rBC 0.09) of fresh CAST soot were minor even when the air-to-fuel ratio was low. ρ_eff_ of fresh CAST soot decreased with increasing size in accordance with the power law (eq 1) and was in the range of 0.3–1.08 g cm^–3^ in the size range of 90–470 nm (Figure 1a). The measured size dependency function was roughly similar to the stove emissions and agrees with previous assessments of fresh CAST particle densities^65^ and soot particles from diesel engines.^30^ (Effective density results of fresh air-starved CAST-soot are not available.)

Similar to the residential combustion emissions, photochemical processing induced a substantial densification of the CAST-emitted particles in the size range of ∼100–500 nm in the mobility diameter. However, the effective size dependency still remained for particles larger than 100 nm. In line with the measured density size dependency, qualitative TEM micrograph analysis displayed that the aged CAST particles were more compact than the fresh particles (Figure 1a,c). For particles smaller than 100 nm, the density did not obey the power law. A possible reason for this is that APM may underestimate the mass of particles smaller than approximately 50 nm^66^ or that the smallest particles have a different composition due to nucleation of organic precursor gases. Under standard combustion conditions, no significant amounts of SOA precursors were emitted from the CAST burner; therefore, organic coating formation was also low. In contrast, under air-starved conditions, photochemical aging notably increased the CF of CAST soot (to 1.56) due to the relatively high amounts of aromatic SOA precursors in the sample, as measured by SPI-ToF-MS (Table 1). The density of the smallest aged CAST particles (*d*_me_ ≲ 80 nm) reached a nearly constant value of 1.6 g cm^–3^, which implies that the structure of these small particles was relatively closed. In addition, the fitted density curve of the fresh particles approaches roughly the same density as the smallest aged particles analyzed. However, the number of particles with *d*_me_ ≲ 80 nm was notably lower in the fresh CAST aerosol than that after aging. This difference may arise from nucleation of the condensable vapors during photochemical processing when primary particle concentrations are sufficiently low.^50,67^ Therefore, it is likely that the particles with *d*_me_ ≲ 80 nm do not originate from the fresh particles, which causes a difference in the density relationship in the smaller size range. The material density of aged CAST OA was estimated to be 1.8 g cm^–3^, which agrees especially well with the sub 80 nm particles, supporting the notion that these smallest particles are indeed formed from nucleation of the organic vapors. The organic coating on the aged soot was also notably more oxidized and estimated to be twice as dense as the fresh, hydrocarbon-like CAST OA.

### Measured and Modeled Aerosol Optical Properties

Light absorption of the exhaust particles was measured online by the aethalometer. In addition, three cases were modeled using an N-Mie core–shell model: fresh CAST, fresh brown coal, and aged brown coal exhaust, representing soot aerosol with negligible coating, mild coating, and thick coating, respectively. All coatings were assumed to be completely scattering in the model.

The changes in the measured AAE (AAE_meas_) upon photochemical processing varied for the three different combustion sources investigated in this work. For wood combustion, photochemical aging did not impact the exhaust AAE_meas_, which were 1.22–1.27 in either fresh or aged aerosols. Such values agree well with the previously measured AAEs of fresh logwood-fired stove emissions,^68,69^ but the lack of increase in AAE upon aging is in contrast to some previous studies, where photochemical processing has been found to increase AAE.^70,71^ Fresh particles from flaming BCB combustion, however, had a rather high AAE_meas_ of 1.75, while the AAE modeled (AAE_mod_) assuming completely scattering coating was 1.19, indicating notable intensification in the absorption in the lower-wavelength region by the UV-light-absorbing BrC species. The AAE_meas_ of the aged BCB particles (1.41–1.49) was lower than the AAE_meas_ in the fresh BCB exhaust, despite the thicker organic coating. The AAE_mod_ of BCB particles, however, increased to 1.27–1.35 during aging, although the core–shell model assumed a fully scattering coating and neglected potential BrC formation. This increase in AAE_mod_ is fully attributed to optical lensing. The measured decrease in AAE during the aging process can be a result of several phenomena. First, photochemical aging may cause decomposition of organic BrC chromophores (so-called photobleaching). For example, extended photochemical aging is known to decompose nitroaromatic compounds and polycyclic aromatic hydrocarbons, which are both known constituents of BrC.^72−74^ Second, compaction of soot agglomerates, as evidently occurred in our experiments, is likely to decrease AAE.^75^

The AAE_meas_ of fresh CAST soot particles produced close to stoichiometric conditions which were close to unity, resembling the often-assumed light absorption of pure mature soot, while the AAE_mod_ for fresh CAST soot assuming core–shell structure was slightly higher (1.19). Under these conditions (λ = 1.05 or 0.98), AAE_meas_ remained close to 1 even after photochemical processing. CAST soot generated under air-starved conditions exhibited notably high AAE_meas_ for both fresh (1.73–1.87) and photochemically processed (1.77) particles, indicating the presence of brown carbon.^76^ A likely explanation is that quenching of the flame under air starved conditions of the CAST burner led to the formation of soot particles with low maturity that contain BrC-like chemical and light absorptive properties, as proposed by Saleh et al. (2018).^77^ The AAE_mod_s can be considered lower limits for AAE of coated soot particles, which would be higher in the case of the coating material containing BrC with a wavelength-dependent imaginary refractive index, instead of the completely scattering coating assumed in the model. Moreover, atmospheric aging may add to light absorption by inducing internal mixing of soot, which amplifies the optical lensing effect.^78^ The core–shell model assumes spherical particles, and the in reality agglomerated structure of the fresh CAST soot may have caused the lower AAE_meas_ compared to AAE_mod_ s. This is supported by the fact that by assuming that the aerosol consists of an external mixture of absorbing BC primary particles, the Mie calculations resulted in a similar AAE (1.03) as measured (Supporting Information, Table S3). However, it is not possible to fully distinguish between the effects of particle morphology and chemical properties on wavelength-dependent light absorption by means of an aethalometer. Furthermore, the aethalometer results are also affected by the loading status of the filter: a full aethalometer filter can be considered to capture the optical properties of bulk aerosol material, while a relatively clean filter may represent single-soot particle properties.^79^

**Figure 2:**
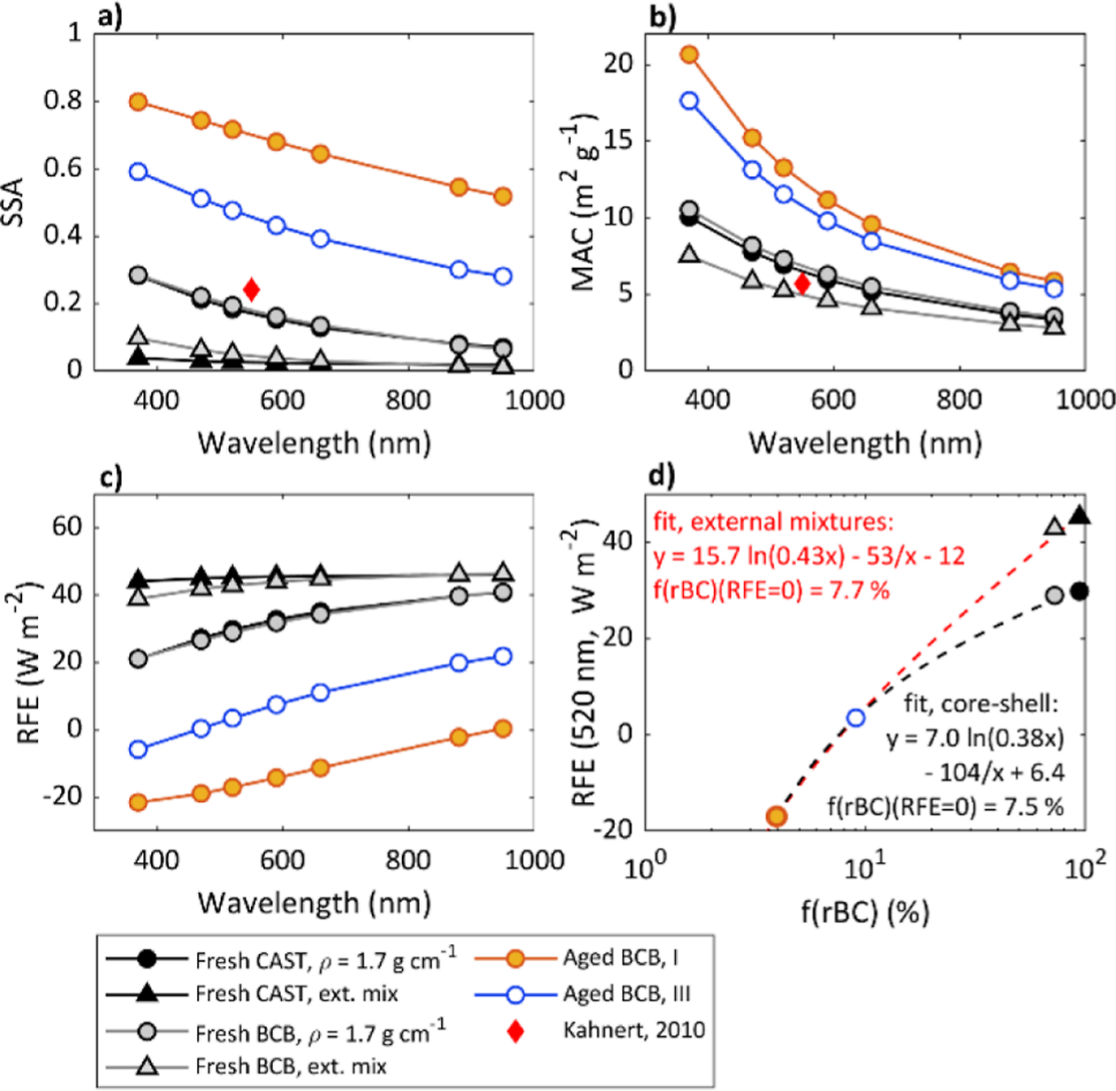
Aerosol optical properties calculated with the core–shell N-Mie model at seven aethalometer wavelengths. (a) SSA, (b) MAC, (c) RFE, and (d) RFE of green light (wavelength 520 nm) as a function of f(rBC). The dashed lines in (d) are empirical fits through the data points.

The modeled optical properties of the fresh exhausts were essentially similar for BCB and CAST aerosols, although the size distribution of rBC from the CAST was much narrower than the size distribution of rBC of the fresh BCB emissions (Supporting Information, Figure S5). Photochemical processing, however, altered all the modeled aerosol optical properties (Figure 2). Namely, the MACs increased due to enhancement in the optical lensing of the radiation to the highly absorbing soot core. Simultaneously, scattering coefficients increased, resulting in a net increase in the single-scattering albedo (SSA) of the aerosol. The increase in both MAC and SSA agrees with previous assessments of optical properties of aging soot.^35,40,80^ The modeled SSA values are consistent with the literature values of SSA (approximately 0.2 ± 0.1 for fresh pure BC and higher for aged aerosols^2^). The core–shell model, however, neglects internal multiple scattering, which would increase upon particle compaction.^81^ It should also be noted that by simply using SP-AMS size distribution data for Mie core–shell modeling, the light absorption is likely slightly overestimated, as indicated by the comparison of MACs between the core shell assumption, soot particle external mixture assumption applying Rayleigh–Debye–Gans theory, and the modeling study of Kahnert (2010)^82^ (Figure 2b). Therefore, knowing soot primary particle sizes and size-dependent effective densities is important to correctly model soot optical properties.

### Radiative Forcing Efficiencies

RFEs at 520 nm were 29.8 and 29.0 W m^–2^ for fresh CAST and BCB exhaust, respectively, and −17.0 or 3.5 W m^–2^ for aged BCB exhaust when assuming core–shell soot structure (Figure 2d). For the external mixture assumption applying Rayleigh–Debye–Gans theory, the RFE values are overestimated because small soot primary balls scatter light less efficiently as real agglomerates (Figure 2a), whereas the MAC values match close to a previous estimate of soot agglomerate by Kahnert (2010).^82^ Nevertheless, all calculated RFE values are high compared to typical atmospheric aerosols with a negative RFE of approximately −25 ± 5 W m^–2^.^83^ Even though the modeled RFE of combustion particles decreased with aging, as expected, the results suggest that the mass fraction of the scattering coating should exceed ∼90% of the total particle mass to allow the particles to become cooling. This value was estimated by an empirical fit to the RFE vs rBC mass fraction f(rBC) (Figure 2d), showing that for RFE to become negative, f(rBC) should decrease to less than ∼7.5%. However, it should be noted that this estimate is based on assumption of a simple core–shell structure and a purely empirical fit to four data points only. Additional measurements of the light absorption, scattering, and coating thickness combined with assessments of soot agglomerate compaction during different states of photochemical and dark aging are needed for more accurate estimates.

### Climate and Health Implications

In this study, we show that photochemical aging strongly affects the ρ_eff_ and mass-mobility relationships of residential combustion particles when encountered with high concentrations of precursors for secondary aerosol formation. So far, several studies have reported that simulated atmospheric aging induces changes in the morphology of laboratory-generated soot.^34,35,38,39,84^ By quantifying the impact of photochemical processing on the morphology and size dependency of the ρ_eff_ of the stove-emitted particles, we show the same phenomena to be important for real-world residential emissions. Thus, the densities and morphologies of residential combustion emissions accompanied by secondary aerosol precursors, or any soot emissions released into polluted urban air, will be subject to considerable changes in the atmosphere as a result of coating formation and compaction.

Wavelength-dependent light absorption and scattering depend on properties such as particle size, agglomerate structure, and thickness and composition of the coating on soot. All these properties are altered by photochemical aging, and detailed measurements at different states of atmospheric exposure are required to comprehensively assess the direct radiative forcing of combustion-derived particles. Here, we show that photochemical aging decreases the direct radiative forcing caused by residential combustion emissions, where soot is often accompanied by relatively high concentrations of secondary aerosol precursors. The morphology of particles has previously been shown to affect the optical properties of particles mainly by altering SSA, while MAC depends more on the properties of the primary particles in the agglomerate than on the agglomerate structure.^13,14,85^ Coating on soot particles, however, generally increases MAC by enhancing optical lensing,^10^ and this enhancement has been noted to be greater for compact soot than for lacy soot.^86^ Thus, compaction upon aging would increase the impact of additional secondary coating formation on particle absorption. Particle compaction may also enhance cloud formation and consequent scattering in the atmosphere, although the link between morphology and cloud condensation activity is not yet fully discerned.^87^ While we show the coating formation as the main driver for residential combustion soot restructuring, the structure of the particles may be further compressed by atmospheric evaporation of the coating, as reported in recent studies.^36,37^ Overall, there is a clear need to further study how the detailed morphological properties of residential combustion particles vary under a wider continuum of atmospheric conditions and to link them with particle optical and hygroscopic properties.

ρ_eff_ is directly linked with the aerodynamic diameter describing particle dynamics in gas flow, and the observed changes would also impact the particle deposition efficiencies in the human respiratory tract. The deposition efficiency of inhaled particles is especially influenced by the effective density for particles above 100 nm. Therefore, using the measured size-dependent particle effective density, instead of unit mass density, is recommended for estimating lung deposition of soot aerosols.^5^ We clearly show that, in the reality, the density of residential combustion particles depends not only on their sizes but also changes in relation to atmospheric processing. We stress that such differences in assumptions need to be accounted for in future studies, for example, in lung deposition models. The compaction of the initially agglomerated structures during photochemical aging also decreases the available surface area, which is an important factor concerning the health effects induced by inhaled particles.^12^ Furthermore, the formation of a hygroscopic organic coating on the initially hydrophobic soot upon atmospheric processing would induce particle growth and consequent restructuring in humid lungs.^11^

We provide, to our knowledge, a first-time quantification of the size dependency of the ρ_eff_ of photochemically aged residential combustion particles. While the ρ_eff_ of fresh combustion aerosols was highly size-dependent, even a minor condensation of organic matter on the surface significantly altered the particle morphology. The change in the size dependency was source-dependent and influenced especially by coating formation, which was strongly linked to the amount of gaseous precursors in the primary exhaust. Since condensational growth is relative to the available surface area, small particles receive a relatively higher mass fraction of condensable matter than larger particles. In addition, the relative thickness of the coating depends on the particle size. For smaller particles, even a minor condensation onto the surface and voids may significantly alter the morphology and produce somewhat closed particles. Initially larger particles, however, would require more condensable material for a measurable change in shape. The findings highlight the importance of understanding the changes in morphology during atmospheric aging of residential combustion particle emissions to correctly capture their behavior in climate and human systems.

## Supporting Information Available

Used experimental setup and conditions, details on the measurement methods, properties of the measured organic aerosols, description on the modeling of the aerosol optical properties, and used definitions of the particle effective density (PDF)
Viewing of fresh and aged brown coal combustion particles in the electron transmission microscope (MP4)
