# Supplementary material for: Photochemical Aging Induces Changes in the Effective Densities, Morphologies, and Optical Properties of Combustion Aerosol Particles
Source: Environ Sci Technol. 2023 Mar 21;57(13):5137–48. doi: 10.1021/acs.est.2c04151 (PMC10077587; doi:10.1021/acs.est.2c04151)
Supplement: Supplementary file 1 — es2c04151_si_001.pdf [file es2c04151_si_001.pdf]

Supporting information for

Photochemical aging induces changes in the effective densities, morphologies and optical properties of combustion aerosol particles

by Leskinen J., Hartikainen A., Väätäinen S., Ihalainen M., Virkkula, A., Mesceriakovas A., Tiitta P., Miettinen M., Lamberg H., Czech H., Yli-Pirilä P., Tissari, J., Jakobi, G., Zimmermann, R., Sippula O.

Supporting information contains

Number of pages: 19

Number of tables: 3

Number of figures: 9

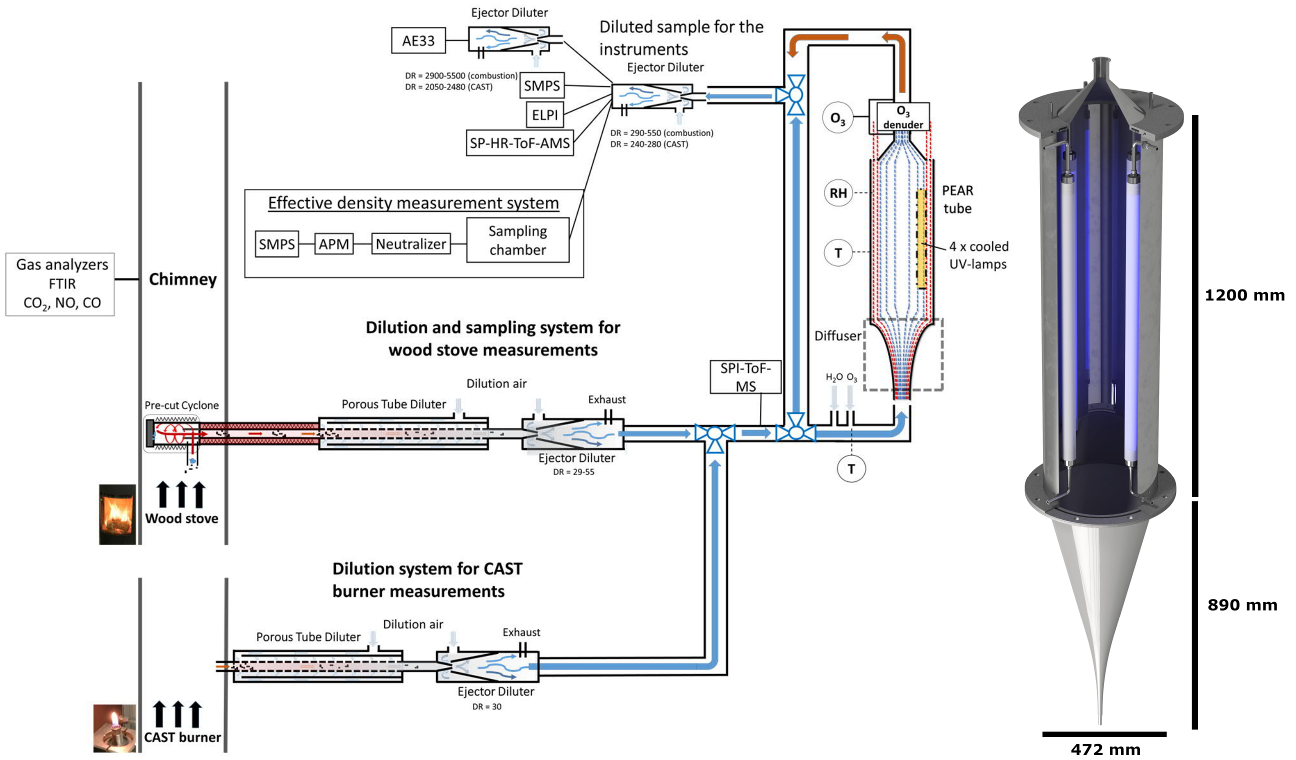

Supplementary Figure S1. Schematics of the experimental setup used for the measurements of aerosol properties. On the right side, the main dimensions of the PEAR tube are shown. DR = dilution ratio.

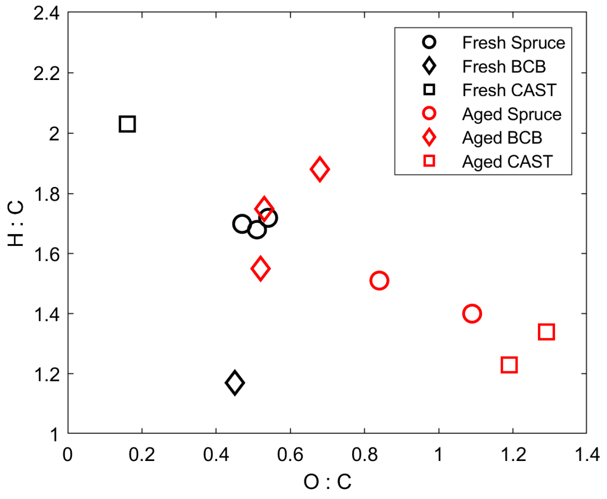

Supplementary Figure S2. Elemental ratios of organic aerosol in the sampled exhausts, measured by SP-HR-ToF-AMS.

S-1. Single-photon ionisation time-of-flight mass spectrometer measurements.

To estimate the amount of SOA precursors available in the exhaust samples, an untargeted analysis for volatile organic compounds (VOCs) was conducted and a selection of aromatic VOCs were semi-quantified from diluted fresh exhaust using a single-photon ionisation time-of-flight mass spectrometer (SPI-TOFMS; compact time-of-flight mass spectrometer II, Firma Stefan Kaesdorf, Germany; Nd:YAG laser: Spitlight400, InnoLas GmbH, Germany) (Czech et al. 2016) . Briefly, in the SPI-TOFMS VOCs get hit by laser radiation of 118 nm, equivalent to 10.49 eV of photon energy. In principle each VOC with a lower ionization energy than this can be ionized. The formed ions are detected by a TOFMS with a mass resolution of approximately 1,000 at m/z 92 and a limit of detection (S/N=3) of approximately 10 ppb toluene at 1 s time resolution. Acquired mass spectra are available in Fig. S3.

The semi-quantification is based on the relative photoionization cross sections (PICS) of VOCs, as described in Adam & Zimmermann (2007), for which we used isotope-labelled toluene (toluene-D3, m/z 95) as internal standard. The true concentration of the internal standard was obtained from comparison to 1 ppm calibration gas containing benzene, toluene, p-xylene and 1,2,4-trimethylbenzene (Linde AG, Germany). A list of targeted VOCs and their PICSs relative to toluene can be found in Table S1. If no PICS could be found in the literature, a PICS of 1 was used as suggested for aromatic compounds by Adam & Zimmermann (2007). The presented concentrations may contain uncertainty up to ±70% for the aromatic VOCs with PICS taken from literature. Furthermore, the results can be considered as upper concertation limits due to possible isobaric compounds. However, all of the selected compounds most likely represent the main contributor to the respective m/z.

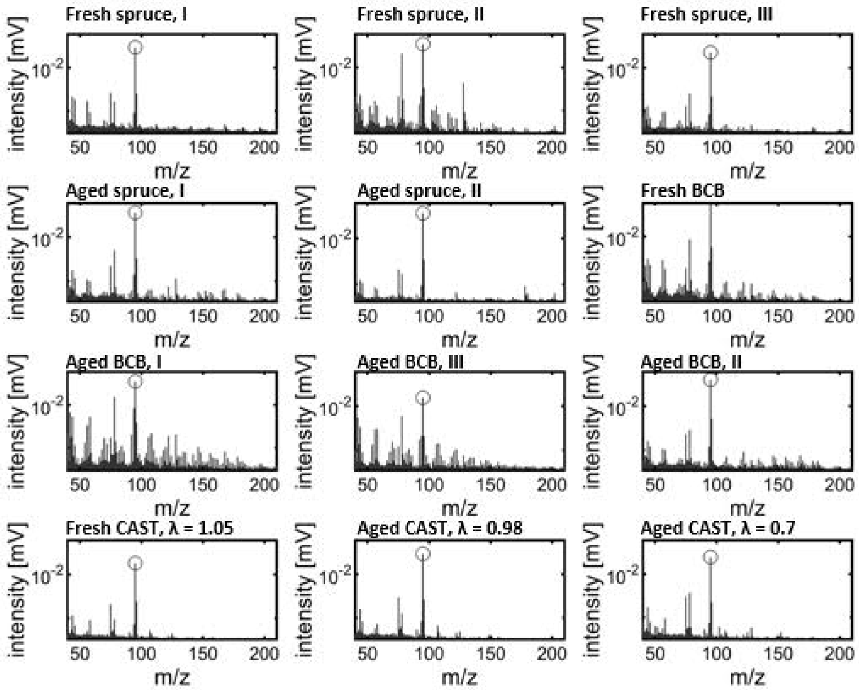

Supplementary Figure S3. Averaged SPI-TOFMS mass spectra of the organic gaseous content during the APM-SMPS measurements. 'o' indicates the internal standard (D3-toluene).

Table S1. Relative photoionization cross sections (PICS) used for the semi-quantification of the aromatic VOCs.

Compound	m/z	Rel. PICS	reference
benzene	78	1.177	Calibration gas
toluene	92	1	Calibration gas
phenol	94	1	Adam & Zimmermann 2007
phenylacetylene	102	1	Adam & Zimmermann 2007
styrene	104	1.329	Adam & Zimmermann 2007
xylene	106	0.962	Calibration gas
methylphenol	108	1	Adam & Zimmermann 2007
indene	116	1.961	Zhou et al., 2009
indane/benzofuran	118	1.147	Zhou et al., 2009
C3-benzene	120	1.258	Calibration gas
dimethylphenol	122	1	Adam & Zimmermann 2007
naphthalene	128	1.118	Adam & Zimmermann 2007
methylnaphthalene	142	1.924	Zhou et al., 2009
acenaphthylene	152	1	Adam & Zimmermann 2007

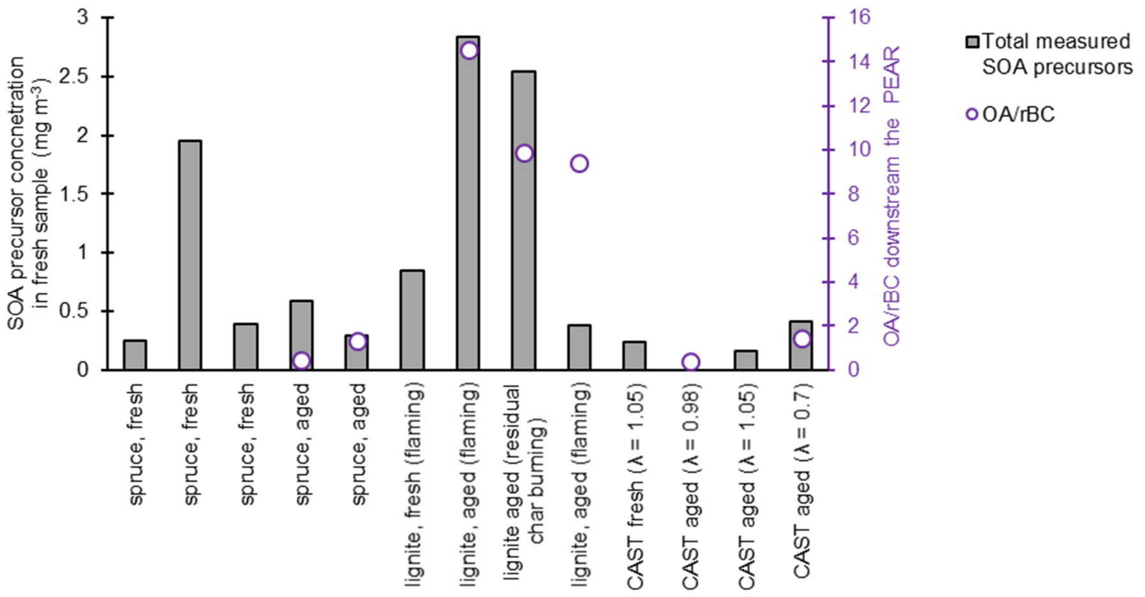

Supplementary Figure S4. Concentrations of the aromatic VOCs measured by SPI-TOFMS from the fresh, diluted exhaust entering the PEAR (not available for CAST aged λ = 0.98), and the ratio of OA to rBC in the photochemically processed exhaust downstream the PEAR.

S-2 Particle effective density and its measurement.

ρ_{eff} (DeCarlo, 2004) is operationally defined based on the instrumentation in use. Different methodologies may lead to results that are not directly comparable, and thus understanding how ρ_{eff} is derived is crucial. Here, ρ_{eff} presents the mass of a single particle (m_p, determined by Aerosol Particle Mass analyser (APM)), divided by volume of a sphere with similar measured electrical mobility diameter (d_{em}, determined by a Scanning Mobility Particle Sizer (SPMS)) (Eq. 1).

$$\rho_{eff} = \frac{6m_p}{\pi d_{em}^3} \qquad \text{(S1)}$$

ρ_{eff} is related to mass-mobility exponent (D_{fm}) via the power law (Eq. 2) Park et al. (2003), which partly illustrates the size-dependency of the particle morphology.

$$\rho_{eff} = K d_{em}^{D_{fm}-3} \qquad \text{(S2)}$$

where d_{em} = electrical mobility diameter, K = constant. Constant ρ_{eff} corresponds to spherical particles, which have a mass-mobility exponent of 3. Mass-mobility exponent is also directly related (but not equal to) the fractal dimension of an agglomerated particle, which describes the morphology depending on the particle's radius of gyration, primary particle size, and the number of primary particles in the agglomerate.

The uncertainties of the effective density measurements were calculated by using the error propagation law assuming ±3% error for the particle size (Kinley & Pui, 1991) and ±10% for the measured mass / charge ratio.

For each measurement to determine the particle effective density as the function of particle size, a sample of diluted flue gas was directed to a custom-made metallic stabilizing chamber with 60 dm^3 volume. The sampling chamber was used to reach a steady sample for the APM-SMPS system because the inherent fluctuation of batch combustion emission prevents direct measurement. Each sampling lasted for 4-13 min, during which a suitable particle number concentration for the APM-SMPS measurement was reached. The APM-SMPS then sampled 1 lpm from the chamber for a period of 44-110 min depending on the concentration of particles in the sampling chamber. The sample was guided through an aerosol neutralizer prior to the APM-SMPS system to achieve a known charge distribution for the particles. The electrical mobility diameter of the mass-classified particles was determined by extracting the geometric mean diameter (GMD) from a log-normal distribution fitted to the measured number size distribution. The GMD was considered the diameter of the spherical particle and used for the volume calculation. There were altogether 5 APM-SMPS measurement runs with spruce combustion, 3 with brown coal and 4 with the CAST burner.

S-3. Modelling of the aerosol optical properties

The aerosol optical properties were modeled for the fresh CAST, fresh BCB, aged BCB I and aged BCB III experiments using a core-shell Mie model at the seven Aethalometer wavelengths (370, 470, 520, 560, 660, 880, and 950 nm) to compare with the AE33 data. This enables us to assess the role of soot core size and its coating thickness and composition on the measured light absorption. A more realistic modeling of optical properties of black carbon fractal aggregates (BCFAs) should be done by using T-matrix codes

(Mishchenko et al., 2004; Kahnert, 2010a, 2010b; Mackowski and Mishchenko, 2011; Romshoo et al., 2021). Such was not available for the present study.

Absorption and scattering coefficients were calculated from

$$\sigma_a(\lambda) = \int Q_a(\lambda, D_p, m_{core}, m_{shell}, s) \tfrac{\pi}{4} D_p^2 n(D_p) dD_p$$
$$\sigma_s(\lambda) = \int Q_s(\lambda, D_p, m_{core}, m_{shell}, s) \tfrac{\pi}{4} D_p^2 n(D_p) dD_p \quad \text{(S3a)}$$

which, for discrete data, are the sums

$$\sigma_a(\lambda) = \sum_i Q_{a,i}(\lambda, D_{p,i}, m_{core,i}, m_{shell,i}, s_i) \tfrac{\pi}{4} D_{p,i}^2 N(D_{p,i})$$
$$\sigma_s(\lambda) = \sum_i Q_{s,i}(\lambda, D_{p,i}, m_{core,i}, m_{shell,i}, s_i) \tfrac{\pi}{4} D_{p,i}^2 N(D_{p,i}) \quad \text{(S3b)}$$

where Q_a and Q_s are the absorption and scattering efficiencies that are functions of the wavelength λ, the diameter D_p, the complex refractive indices of the core and shell, m_{core} and m_{shell}, respectively, and the shell thickness s for the particle number size distribution $n(D_p)$. For calculating Q_a and Q_s, a N-Mie Fortran code (Voshchinnikov and Mathis, 1999) based on a recursive algorithm of Wu and Wang (1991). The code was also used by Virkkula (2021) for evaluating the effect of coating BC particles with purely scattering material on the absorption Ångström exponent and subsequent source apportionment.

The particle size distributions of refractory black carbon (rBC), organics, ammonia, sulphate, and nitrate were measured by the SP-HR-ToF-AMS. The aerodynamic particle diameter range of the size distributions is 11.68 – 4351.6 nm, in 118 size bins. For simplicity it was assumed that inorganics and organics form a mixed purely light-scattering shell on the rBC core at each particle diameter measured by the SP-AMS. The applied complex refractive indices were m_{core} = 1.85 + 0.71i (refractive index of BC as in Kahnert, 2010b, and Lack and Cappa, 2010) and m_{shell} = 1.52+0i (ammonium sulfate) for the core and shell, respectively. However, for the aged BC data with high coating also a three-layer sensitivity study was conducted by assuming that the shell consists of an inorganic layer with $m_{shell1,inorg}$ = 1.52+0i and an organic layer with three different values $m_{shell2,org}$ = 1.50+0i,1.52+0i and 1.54+0i. The resulting differences were minimal: for instance, for the aged BCB I experiment, the single-scattering albedo at λ = 520 nm was 0.709, 0.717 and 0.724, respectively, for the three different organic layer refractive indices. The differences were so small that in the rest of the paper only one refractive index, m_{shell} = 1.52+0i, for the shell is used. The core and shell thicknesses were calculated from the SP-HR-ToF-AMS data for each of the 118 diameters.

The SP-AMS data are presented as mass size distributions as a function of aerodynamic diameter D_a. The scattering and absorption calculations were done by using the diameters of Stokes equivalent spheres:

$$D_p = D_a \rho_p^{-1/2}, \quad \text{(S4)}$$

where ρ_p is the particle density. The number concentrations were calculated from

$$N(D_{p,i}) = \frac{6m_i}{\pi\rho_i D_{p,i}^3}, \quad (S5)$$

where m_i is the mass concentration in the particle diameter bin i of the SP-AMS. For the fresh CAST and the fresh BCB data ρ_p was strongly size dependent (Fig. 1) and it was presented with the power law (S2)

S2 and S4 yield the equation

$$D_p = D_a^{\frac{2}{D_{fm}-1}} K^{-\frac{1}{D_{fm}-1}}. \quad (S6)$$

However, the effective densities of fresh CAST and fresh BCB particles were clearly lower than 1 g cm^{-3}, and according to Eq. (S2), decrease with increasing particle size. This leads to widening of the size distribution and to larger particle diameters. Consequently, the aerosol optical properties calculated by the Mie core-shell modeling became unrealistic for fresh BC. For instance, for the fresh CAST and BCB data, the mass absorption coefficient at $\lambda =$ 520 nm were 16.2 m^2 g^{-1} and 19.5 m^2 g^{-1} for fresh CAST and fresh BCB, respectively, which is more than twice as high as that presented by Bond and Bergstrom (2006) and Kahnert (2010b).

To capture the optical properties of fractal soot particles, the Mie calculations for the fresh CAST and BCB data were conducted using three approaches: 1) the above-described variable-density calculations for the mass size distributions obtained from the SP-HR-ToF-AMS and applying the core-shell model, 2) by using a constant density ρ_p = 1.7 g cm^{-3} for the mass size distributions obtained from the SP-HR-ToF-AMS and the core-shell model and 3) by assuming that the aerosol consists of an external mixture of scattering organic and inorganic particles and absorbing BC particles.

In approach 1, the fact that ρ_p < 1 g cm^{-3} and that it decreases the larger the particles are, leads to widening of the size distribution and to larger particle diameters. Consequently, the aerosol optical properties calculated by the Mie core-shell modeling became unrealistic for fresh BC. For instance, for the fresh CAST and BCB data, the mass absorption coefficient at $\lambda =$ 520 nm were 16.2 m^2 g^{-1} and 19.5 m^2 g^{-1} for fresh CAST and fresh BCB, respectively, which is more than twice as high as that presented by Bond and Bergstrom (2006) and Kahnert (2010b). This approach was therefore considered the most uncertain and will not be discussed further.

The reasoning for using approach 2 is that if only size distribution data from an SP-AMS were available, it is logical to use them for core-shell Mie modeling, especially when the size distributions look like those in Figs. S5a and S5b where the size distributions of both rBC and organics are relatively narrow and their geometric mean diameters are the same. This is probably the most typical use of size distributions for modeling scattering and absorption. The reasoning for using the density 1.7 g cm^{-3} in this approach is that the density of BC primary spheres recommended by Bond and Bergstrom (2006) is 1.8 g cm^{-3} and there is also organics that lower the density.

In approach 3 (external mixture), it was assumed that that the BC agglomerates consist of primary spheres and Rayleigh–Debye–Gans (RDG) theory was applied. The RDG theory

considers the primary particles in the aggregate to be individual Rayleigh scatterers, and the inter-particle scattering is ignored (Sorensen, 2001; Romshoo et al., 2021). Consequently, in the RDG theory, the total light absorption by the aggregate is the sum of light absorption by individual primary particles. For this purpose, lognormal number size distributions $n_{BC}(D_g,\sigma_g,N_{tot})$ of primary BC spheres were generated where D_g, σ_g and N_{tot} are the geometric mean diameter, geometric standard deviation and the total number concentration, respectively. Bond and Bergstrom (2006) use the term primary monomer radius and state that it typically varies in the range of 10-25 nm, which corresponds to the diameter range of 20-50 nm. Therefore, the primary BC-sphere size distributions were first given the values D_g = 20, 30, 40, 50 and 60 nm and σ_g = 1.3. The density of the spheres was set to ρ_p = 1.8 g cm^{-3}, as recommended by Bond and Bergstrom (2006). The figures in the text were plotted from the smallest of these, D_g = 20, as this is the most realistic assumption of primary particle size based on the TEM figures (Fig. 1). Results achieved with the larger D_g values are, however, presented in the Table S3. N_{tot} was varied so that the number size distribution calculated from the BC mass concentration matched the total rBC mass concentration measured with the SP-HR-ToF-AMS. Scattering and absorption by BC was then calculated from Eqs. (S3). The scattering by the organic and inorganic particles measured with the SP-HR-ToF-AMS were calculated from Eqs. (S3) – (S5) assuming that the density was 1.4 g cm^{-3}.For the aged BCB data the density did not vary significantly with size (Fig. 1), and the densities ρ_p = 1.4 g cm^{-3} and ρ_p = 1.7 g cm^{-3} were used for the calculations for aged BCB I and BCB III, respectively, in agreement with Fig. 1.

The calculated σ_s and σ_a were used for calculating the single-scattering albedo (SSA).

$$SSA = \frac{\sigma_s}{\sigma_s+\sigma_a} \quad \text{(S7)}$$

The code also calculates the asymmetry parameter g, which describes the angular distribution of scattered light:

$$g(\lambda,m) = \frac{1}{2}\int_0^{\pi} P(\theta,\lambda,m)\sin\theta\cos\theta\ d\theta \quad \text{(S8)}$$

where P is the scattering phase function and θ is the scattering angle. If $g > 0$, light is scattered forward ($g = +1$ for entirely forward scatter), for $g = 0$ scattering is isotropic (i.e., even to all directions), and for $g < 0$ light is backscattered (-1 for entirely backscattered light). Particles that scatter symmetrically have an asymmetry parameter of zero, while particles with more forward scattering, i.e., large particles, approach asymmetry parameter values of 1. From *g* it is possible to estimate the average upscatter fraction β, which is one of the properties controlling the aerosol direct radiative forcing (e.g., Andrews et al., 2006, Moosmüller and Ogren, 2017). The larger β is, the more aerosols scatter light to space and cool the atmosphere, or heat it less in the case of aerosol so dark that it heats the atmosphere. Radiative forcing efficiencies (RFE = $\Delta F/\tau$) at the top of the atmosphere, i.e., aerosol forcing per unit optical depth (τ), were calculated using formula (S9), which is based on equations and constants presented by Haywood and Shine 1995, Sheridan and Ogren 1999 and Delene and Ogren 2002.

$$\frac{\Delta F}{\tau} = -DS_0T_{at}^2(1-A_c)\omega_0\beta\left\{(1-R_s)^2-\left(\frac{2R_s}{\beta}\right)\left[\left(\frac{1}{\omega_0}\right)-1\right]\right\} \quad \text{(S9)}$$

where D is the fractional day length, S_o is the solar constant, T_{at} is the atmospheric transmission, A_c is the fractional cloud amount, and R_s is the surface reflectance. The non-aerosol-related factors were kept constant: D = 0.5, S_o = 1370 W m^{-2}, T_{at} = 0.76, A_c = 0.6, and R_s = 0.15, as suggested by Haywood and Shine (1995). β was assumed to have no zenith angle dependence and was calculated from equation (S10) (Moosmüller and Ogren, 2017).

$$\beta = -0.2935\, g^3 + 0.2556\, g^2 - 0.4489\, g + 0.5043. \qquad \text{(S10)}$$

Mass absorption coefficients (MAC) were calculated from

$$\mathrm{MAC}(\lambda) = \frac{\sigma_a(\lambda)}{m(\mathrm{rBC})} \qquad \text{(S11)}$$

where m(rBC) is the mass concentration of rBC. Modelled absorption Ångström exponents (AAE) were calculated from Eq. S12 using the calculated absorption coefficients.

$$\mathrm{AAE} = -\frac{\ln\left(\frac{\sigma_a(\lambda=470\mathrm{nm})}{\sigma_a(\lambda=950\mathrm{nm})}\right)}{\ln\left(\frac{470}{950}\right)} \qquad \text{(S12)}$$

The SP-HR-ToF-AMS size distributions used in the calculations are shown in Fig. S5. In the fresh CAST and brown coal combustion emissions the concentrations of the inorganic compounds were below the detection limits. The integrated rBC concentrations, their mass fractions, and lognormal mode parameters of the rBC size distributions are presented in Table S2.

Table S2. rBC concentrations, mass fractions f(rBC) and lognormal mode parameters of the rBC size distributions in the four size distributions used for the core-shell model and the resulting modelled AAE_{mod} for the wavelength pair 470/950, and the RFE values at 520 nm (shown in Figure 2). Aethalometer based measured AAEs (AAE_{meas}) are given for comparison. $D_{g,a}$: geometric mean aerodynamic diameter, σ_g: geometric standard deviation. For the fresh aerosol the core-shell models were calculated with density 1.7 g cm^{-3} and for the external mixtures with the primary particle sphere size distributions of D_g = 20 nm.

	unit	Fresh CAST		Fresh BCB (D_a < 450 nm)		Aged BCB, I	Aged BCB, III
rBC mass	mg m^{-3}	11377		5125		1776	4062
f(rBC)	%	93.8		77.1		3.95	9.07
$D_{g,a}$(rBC)	nm	127		133		194	191
σ_g(rBC)		1.17		1.33		1.53	1.47
AAE_{meas}		0.94		1.75		1.41	1.46
		core-shell	*ext.mixt.*	*core-shell*	*ext.mixt.*	*core-shell*	*core-shell*
AAE_{mod}		1.19	1.03	1.19	1.03	1.31	1.26
RFE, 520 nm	W m^{-2}	29.8	45.2	29.0	42.9	-17.0	3.5

Table S3. Model results for the external mixture of BC and organic particles. The BC particle agglomerates were assumed to behave in accordance with the Rayleigh–Debye–Gans theory and the organic particle size distributions were assumed to be those presented in Fig. S5a and S5b. Primary BC-sphere size distributions were generated with different geometric mean diameters (D_g(BC)) and geometric standard deviation (σ_g) of 1.3.

		D_g(BC)		
	**Wavelength (nm)**	**20 nm**	**40 nm**	**60 nm**
***MAC,* Fresh CAST and BCB**	370	7.53	8.40	9.28
(m^2 g^{-1})	470	5.82	6.29	6.89
	520	5.24	5.59	6.07
	590	4.59	4.84	5.20
	660	4.09	4.27	4.54
	880	3.04	3.12	3.25
	950	2.82	2.88	2.98
SSA, Fresh CAST	370	0.038	0.094	0.189
	470	0.029	0.059	0.123
	520	0.026	0.049	0.101
	590	0.024	0.040	0.079
	660	0.022	0.034	0.063
	880	0.019	0.024	0.037
	950	0.018	0.022	0.033
SSA, Fresh BCB	370	0.097	0.141	0.224
	470	0.062	0.088	0.146
	520	0.050	0.071	0.119
	590	0.038	0.053	0.090
	660	0.029	0.040	0.069
	880	0.014	0.019	0.033
	950	0.011	0.015	0.027
RFE, Fresh CAST	370	44.10	38.35	29.16
(W m^{-2})	470	45.02	41.85	35.40
	520	45.25	42.84	37.58
	590	45.52	43.80	39.83
	660	45.67	44.43	41.42
	880	45.99	45.44	44.04
	950	46.07	45.61	44.48
RFE, Fresh BCB	370	38.91	34.24	26.18
(W m^{-2})	470	41.86	39.10	33.23
	520	42.86	40.71	35.82
	590	43.98	42.39	38.62
	660	44.77	43.59	40.66
	880	46.12	45.57	44.16
	950	46.38	45.92	44.77
**AAE, Fresh CAST and BCB**	470/950	1.03	1.11	1.19

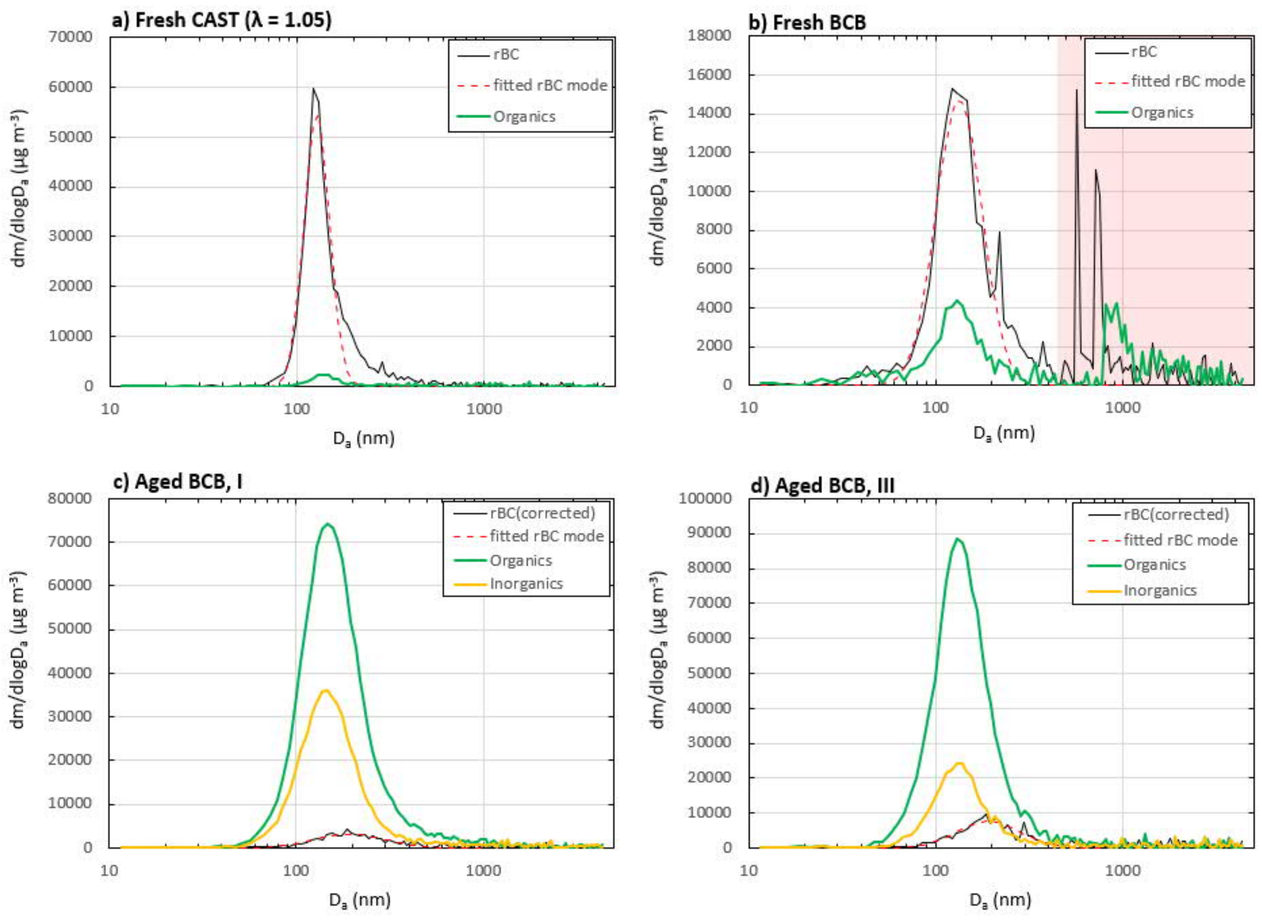

Supplementary Figure S5. The mass size distributions used in the core-shell model calculations, measured by the SP-HR-ToF-AMS. In the size distribution of fresh brown coal combustion emissions the particles in the size range D_a >450 nm (red background in (b)) were omitted from the calculations due to a measurement artifact. The red dashed lines show the lognormal modes fitted with the rBC size distributions.

S-4. Experimental conditions in the PEAR

Approximately 5 ppm of O_3 was fed externally into the PEAR, where it was photolyzed by the UV-radiation centered at 254 nm. This leads to formation of oxygen radicals (O(1D)) and subsequent formation of OH-radicals in the presence of H_2O vapor. Photon flux of 3 × 10^{16} photons cm^{-2} s^{-1} was estimated based on the UV lamp power and efficiency and the PEAR internal surface area (2.28 m^2). The relative humidity in the PEAR was 50 %. The total flow rate through the PEAR was 100 dm^3 min^{-1} for stove experiments and 50 lpm for the CAST experiments, leading to mean residence times of 70 s and 140 s in the PEAR for the stove and CAST experiments, respectively. In this relatively short period, high levels of OH exposure were reached. Integrated OH exposures downstream the PEAR were modelled based on the concentration of reactive gases in the exhaust. (Hartikainen et al. 2020) Median OH exposures during sampling were estimated to reach (0.9 – 2.6) x 10^{11} molec. cm^{-3} s, which correspond to 1 – 4 days of exposure at ambient OH concentration of 10^6 molec. cm^{-3}.

Experimental conditions in the PEAR were assessed similarly to Hartikainen et al. 2020. In short, the fate of gaseous low-volatility organic compounds (LVOCs) in the PEAR was

estimated for stove experiments as in Palm et al. 2016 with particulate condensation sinks (Lehtinen et al. 2003; Fig. S6) based on the particle size distributions measured by SMPS downstream the PEAR. Majority of LVOCs were estimated to condense on the particles, while below 5% was lost to walls during the APM-SMPS stove experiments (Figure S7). In the CAST experiments particulate condensation sink was notably lower, leading to higher relative share of LVOCs condensing to the walls instead. Particulate wall losses in the PEAR are below 2 % (Ihalainen et al. 2019) due to the relatively low area-to-volume-ratio and the ring flow outlets, through which the flow closest to the walls is discarded from further analyses.

External OH reactivity (OHR_{ext}) caused by the gaseous components in the reactor was calculated for the stove experiments based on FTIR measurements (Hartikainen et al. 2020). OHR_{ext} in the PEAR during the stove experiments can be considered relatively high (median reactivity 780 – 2300 s^{-1}; Supplementary Figure S8a). In the CAST experiments gaseous emissions and OHR_{ext} were estimated based on the SPI-measurements and can be expected to be lower than in the stove experiments. Integrated OH exposure downstream the PEAR was modeled based on OHR_{ext} (Peng et al. 2016) (Supplementary Fig. S8b). Photochemical processing was influenced by higher-than-ambient levels of photolysis especially for two lignite experiments (char burning and 9 min after ignition), for which the ratio of photolysis to OH exposure rose above 2×10^7 photons cm s^{-1}.

The photochemical processing in oxidation flow reactors is in general extreme compared to real-life atmospheric transformation. The ratio of photolysis to OH exposure is notably higher than in the atmosphere, which impacts the reaction pathways and for example the fate of the peroxy radicals (RO_2) produced from the reactions of organic gases (Peng and Jimenez, 2020). The relative importance of RO_2 reactions with OH and HO_2 reactions are enhanced, while RO_2 does not have sufficient time to isomerize in the same extent as in the atmosphere. Despite the differences to ambient air or smog chamber experiments, the fast sample processing of flow reactors makes them useful tools especially for photochemical exposure of dynamic combustion emissions. Furthermore, a comparison a chemical characteristics of combustion aerosol samples processed between with an environmental chamber and PEAR showed a high similarity (Ihalainen et al. 2019).

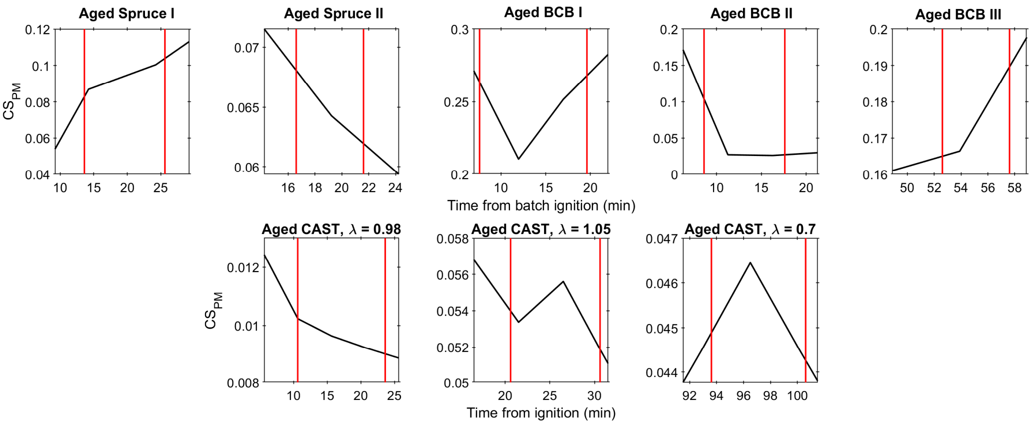

Supplementary Figure S6. Particulate condensation sink (CS_{PM}) downstream the PEAR. Red lines indicate the collection period for the APM-SMPS experiments.

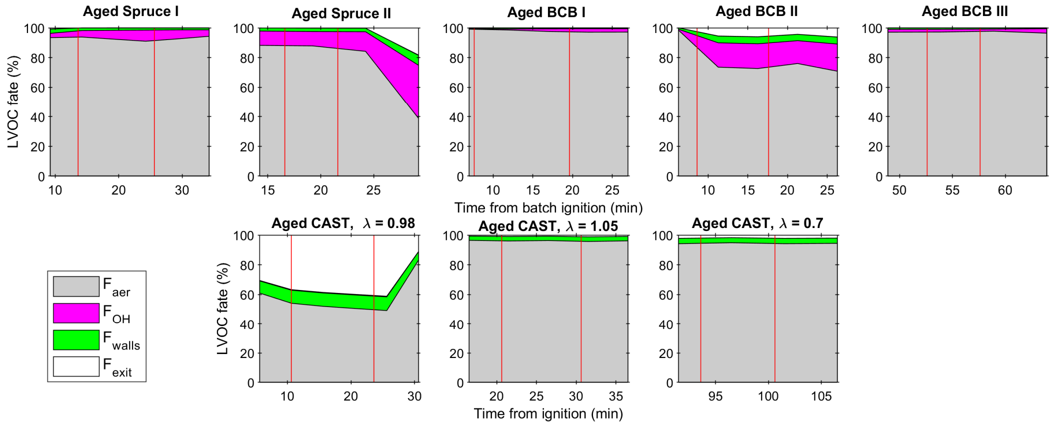

Supplementary Figure S7. Fate of low-volatility organic compounds (LVOCS) with vapor pressures low enough for irreversible condensation to particulate phase (F_{aer} = condensation on to particulate phase; F_{OH} = reacted with OH in the PEAR (not estimated for CAST); F_{walls} = lost to walls; F_{exit}= LVOCs exiting the PEAR in the gas phase). Red lines indicate the collection period for the APM-SMPS experiments.

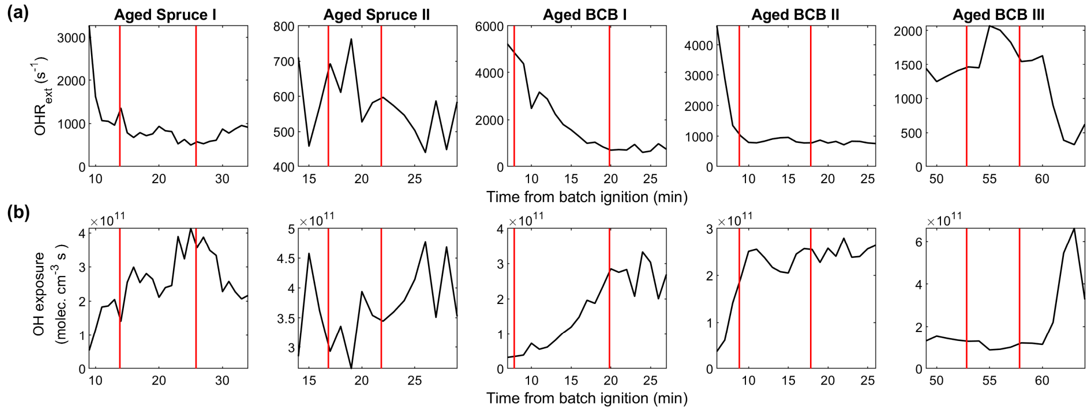

Supplementary Figure S8. Experimental conditions in the PEAR during the stove experiments: external OH reactivity (OHR_{ext}) entering the PEAR during the experiments (a) and integrated OH exposure downstream the PEAR (b). Red lines indicate the collection period for the APM-SMPS experiments.

S-5 Gas analysers.

The emissions of carbon monoxide, carbon dioxide, nitrogen oxides, total organic gaseous compounds, and oxygen were measured for the wood stove directly from the flue gas through an insulated and heated (180 °C) sampling line by single gas analysers (Magnos 106, Uras 14, Limas 11 UV, ABB and Multi-FID 14, ABB). In addition, the gaseous composition of the fresh stove emissions were measured directly from the flue gas through an insulated and heated sampling line (180 °C) by Fourier Transform Infrared spectroscopy (FTIR-DX4000, Gasmet). Moreover, a single-photon ionisation time-of-flight mass spectrometer (SPI-ToF-MS) was used to conduct untargeted analysis and semi-quantification of aromatic VOCs in the both stove and CAST burner experiments (see Supplemenetary section S1). The overall experimental conditions and gaseous emissions

from the stove, including both FTIR and SPI-ToF-MS results, are discussed in detail by Martens et al. (2021).

S-6 Sampling and dilution.

In the stove experiments, exhaust was sampled by an externally heated sampling probe and diluted by a total ratio of approximately 30 by a combination of a porous tube diluter and an ejector diluter (Lyyränen et al. 2004). The sample was then diluted by an additional factor of 10 by another ejector diluter (Palas GmbH DR-10) upstream the online instruments (SP-AMS, SMPS, and ELPI). CAST combustion aerosol was sampled directly after the flame with a high-temperature porous tube sampling probe[47] and diluted by two ejector diluters (Dekati Ltd. FPS-4000 DR-8 and Palas GmbH DR-10) by a total ratio of 240-280 before the online instruments. For both setups, an additional ejector diluter (Palas GmbH DR-10) with a dilution ratio of 10 was in use prior to the aethalometer. Dilution air was purified with a zero-air generator (Model 737-250 AADCO Instruments Inc., USA).

S-7 Soot Particle Aerosol Mass Spectrometer (SP-AMS) measurements.

Particle chemical compositions and coating factors (CFs) were derived by a high-resolution soot particle time-of-flight aerosol mass spectrometer (SP-AMS, Aerodyne Research Inc. (Onasch 2012). The SP-AMS was operated similarly to Hartikainen et al., (2020). The two vaporizer configurations were alternated every 120 s (including the 20 s particle time-of-flight (PTOF) mode). First, the nonrefractory submicron particles (NR-PM1, including organic aerosol (OA), nitrate, sulfate, ammonium, and chloride) were analyzed using the tungsten mode, where the thermal vaporizer was operated at 600 °C. Second, both NR-PM1 and refractory particles (R-PM, namely, refractory black carbon, rBC) were studied using the dual vaporizer mode, with the combination of the thermal vaporizer and the continuous wave laser vaporizer (1064 nm). CF was calculated as the ratio of the total NR-PM1 mass to the rBC mass. Regal Black (Regal 400R Pigment Black, Cabot Corp.) particles were measured in the beginning and end of the campaign to determine the relative ionization efficiency for rBC (Figure S9, RIErBC = 0.24), following the SP-AMS calibration presented by Onasch et al. (2012). Shortly, standard mass-based calibrations were performed for the ionization efficiency in the AMS using dried and size-selected ammonium nitrate (NH4NO3) particles (Jayne et al., 2000). The particles were generated by atomization of a solution of ammonium nitrate or Regal black, dried via a diffusion drier to relative humidity less than ~30%, and size selected (300 nm) by a Differential Mobility Analyzer (DMA TSI model 3081). Collection efficiency (CE) of 1 was applied. rBC was determined using high-resolution analysis of mass spectra to minimize interference by overlapping peaks (updated UMR rBC fragmentation table). Elemental analysis of OA was performed using the improved-ambient method (Canagaratna et al., 2015).

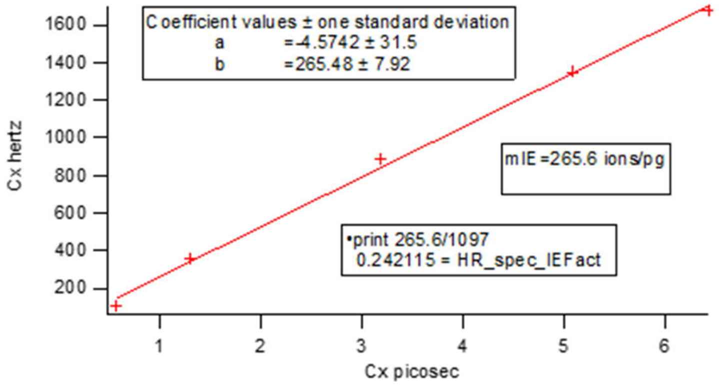

Supplement Figure S9. rBC calibration for SP-AMS.

S-8 Operation of the Combustion Aerosol Standard (CAST) burner

A combustion aerosol standard (CAST) burner (Cast Jing Ltd.) was used for soot particle production using propane gas as fuel. The air-to-fuel ratios (λ) of the CAST combustion were altered to vary the combustion conditions and to generate particles with varying properties. First, the CAST standard settings were used to generate nominally 210 nm particles (fuel 0.055 Lpm, quench gas 7.5 Lpm, oxidation gas 1.4 Lpm, dilution air 20 Lpm, resulting in λ of 1.05). In the air-starved experiments (λ = 0.73 or 0.67), the combustion air availability was limited by reducing the oxidation gas flow to 0.96 Lpm. Propane flow was 0.055 Lpm and 0.06 Lpm in the experiments with λ = 0.73 and 0.67, respectively. The CAST aerosol sample was collected downstream of the initial quenching and dilution in the upper part of the burner but before the rotary disc diluter in the CAST.

References

Adam T., Zimmermann R. Determination of single photon ionization cross sections for quantitative analysis of complex organic mixtures. *Anal Bioanal Chem.* ***2007****, 389(6):1941-1951; DOI 10.1007/s00216-007-1571-x.*

Andrews E., Sheridan PJ., Fiebig M., McComiskey, A., Ogren J.A., Arnott P., Covert D., Elleman R., Gasparini R., Collins D., Jonsson H., Schmid B., Wang J. Comparison of methods for deriving aerosol asymmetry parameter. *J Geophys Res D Atmos.* ***2006****, 111(5); DOI 10.1029/2004JD005734.*

Bond, T. C. and Bergstrom, R. W. Light absorption by carbonaceous particles: An investigative review, *Aerosol Sci. Technol.,* ***2006****, 40, 27–67,* https://doi.org/10.1080/02786820500421521.

Canagaratna M.R.; Jimenez J.L.; Kroll J.H.; Chen Q.; Kessler S.H.; Massoli P.; Hildebrandt Ruiz L.; Fortner E.; Williams L.R.; Wilson K.R.; Surratt J.D.; Donahue N. M.; Jayne J.T.; Worsnop D.R. Elemental ratio measurements of organic compounds using aerosol mass spectrometry: Characterization; improved calibration; and implications. Atmos Chem Phys. 2015, 15(1):253-272; DOI 10.5194/acp-15-253-2015.

Czech H., Sippula O., Kortelainen M., Tissari J., Radischat C., Passig J., Streibel T., Jokiniemi J., Zimmermann R. On-line analysis of organic emissions from residential wood combustion with single-photon ionisation time-of-flight mass spectrometry (SPI-TOFMS). *Fuel.* ***2016****, 177:334-342. DOI 10.1016/j.fuel.2016.03.036.*

DeCarlo, P. F.; Slowik, J. G.; Worsnop, D. R.; Davidovits, P.; Jimenez, J. L. Particle morphology and density characterization by combined mobility and aerodynamic diameter measurements. Part 1: Theory. *Aerosol Sci. Technol.* ***2004****, 38 (12) 1185– 1205*.

Delene D.J., Ogren J.A. Variability of aerosol optical properties at four north american surface monitoring sites. *J Atmos Sci.* ***2002****, 59(6):1135-1150; DOI 10.1175/1520-0469(2002)0592.0.CO;2.*

Haywood J.M., Shine K.P. The effect of anthropogenic sulfate and soot aerosol on the clear sky planetary radiation budget. *Geophys Res Lett.* ***1995****, 22(5):603-606; DOI 10.1029/95GL00075.*

Hartikainen A., Tiitta P., Ihalainen M., Yli-Pirila P., Orasche J., Czech H., Kortelainen M., Lamberg., Suhonen H., Koponen H., Hao L., Zimmermann R., Jokiniemi J., Tissari J., Sippula O. Photochemical transformation of residential wood combustion emissions: Dependence of organic aerosol composition on OH exposure. *Atmos Chem Phys.* ***2020****, 20(11):6357-6378; DOI 10.5194/acp-20-6357-2020.*

Ihalainen M., Tiitta P., Czech H., Yli-Pirilä P., Hartikainen A., Kortelainen M., Tissari J., Stengel B., Sklorz M., Suhonen H., Lamberg H., Leskinen A., Kiendler-Scharr A., Harndorf H., Zimmermann R., Jokiniemi J., Sippula O A novel high-volume photochemical emission aging flow tube reactor (PEAR). *Aerosol Science and Technology.* ***2019****, 53(3):276-294; DOI 10.1080/02786826.2018.1559918.*

Kahnert, M.: Numerically exact computation of the optical properties of light absorbing carbon aggregates for wavelength of 200 nm–12.2 µm. *Atmos. Chem. Phys.* ***2010****a, 10, 8319–8329, https://doi.org/10.5194/acp-10-8319-2010.*

Kahnert, M.: On the discrepancy between modeled and measured mass absorption cross sections of light absorbing carbon aerosols. *Aerosol Sci. Technol., **2010**b, 44, 453–460, https://doi.org/10.1080/02786821003733834.*

Kinney P.D., and Pui D.Y.H.: Use of the electrostatic classification method to size 0.1 µm SRM particles – A feasibility study. *J. Res. Natl. Inst. Stand. Technol.* ***1991*** *96(147).*

Lack D.A., Cappa C.D. Impact of brown and clear carbon on light absorption enhancement, single scatter albedo and absorption wavelength dependence of black carbon. *Atmos Chem Phys.* ***2010****, 10(9):4207-4220; DOI 10.5194/acp-10-4207-2010.*

Lehtinen K.E.J., Korhonen H., Dal Maso M., Kulmala M. On the concept of condensation sink diameter. *Boreal Environ Res.* ***2003****, 8(4):405-411.*

Lyyränen J., Backman U., Tapper U.; Auvinen A., Jokiniemi J. A size selective nanoparticle collection device based on diffusion and thermophoresis. *J Phys Conf Ser.* ***2009****, 170; DOI 10.1088/1742-6596/170/1/012011.*

Mackowski D. W. and Mishchenko M. I. A multiple sphere T-matrix Fortran code for use on parallel computer clusters. *J. Quant. Spectrosc. Radiat. Transf.,* ***2011****, 112, 2182–2192, https://doi.org/10.1016/j.jqsrt.2011.02.019.*

Martens P., Czech H., Tissari J., Ihalainen M., Suhonen H., Sklorz M., Jokiniemi J., Sippula O., Zimmermann R. Emissions of gases and volatile organic compounds from residential heating: A comparison of brown coal briquettes and logwood combustion. *Energy Fuels.* ***2021****,* DOI 10.1021/acs.energyfuels.1c01667.

Mishchenko M. I., Liu L., Travis L. D., and Lacis A. A.: Scattering and optical properties of semi-external versus external mixtures of different aerosol types. *J. Quant. Spectrosc. Radiat. Transf.,* ***2004****, 88, 139–147, https://doi.org/10.1016/j.jqsrt.2003.12.032.*

Moosmüller H., Ogren J.A. Parameterization of the aerosol upscatter fraction as function of the backscatter fraction and their relationships to the asymmetry parameter for radiative transfer calculations. *Atmosphere.* ***2017****, 8(8); DOI 10.3390/atmos8080133.*

Onasch, T. B., Trimborn, A., Fortner, E. C., Jayne, J. T., Kok, G. L., Williams, L. R., Davidovits, P., and Worsnop, D. R.: Soot particle aerosol mass spectrometer: development, validation, and initial application, Aerosol Sci. Technol., 46, 804–817, doi:10.1080/02786826.2012.663948, 2012.

Palm B.B., Campuzano-Jost P., Ortega A.M., Day D.A., Kaser L., Jud W., Karl T., Hansel A., Hunter J.F., Cross E.S., Kroll J.H., Peng Z., Brune W.H., Jimenez J.L. In situ secondary organic aerosol formation from ambient pine forest air using an oxidation flow reactor. *Atmos Chem Phys.* ***2016****, 16(5):2943-2970; DOI 10.5194/acp-16-2943-2016.*

Park K.; Cao F.; Kittelson D.B.; McMurry P.H. Relationship between particle mass and mobility for diesel exhaust particles. *Environ Sci Technol.* ***2003****, 37(3):577-583; DOI 10.1021/es025960v.*

Peng Z., Day D.A., Ortega A.M., Palm B.B., Hu W., Stark H., Li R., Tsigaridis K., Brune W.H., Jimenez J.L. Non-OH chemistry in oxidation flow reactors for the study of atmospheric chemistry systematically examined by modeling. *Atmos Chem Phys.* ***2016****, 16(7):4283-4305; DOI 10.5194/acp-16-4283-2016.*

Peng Z., Jimenez J.L. Radical chemistry in oxidation flow reactors for atmospheric chemistry research. *Chem Soc Rev.* ***2020****, 49(9):2570-2616; DOI 10.1039/c9cs00766k.*

Romshoo, B., Müller, T., Pfeifer, S., Saturno, J., Nowak, A., Ciupek, K., Quincey, P., and Wiedensohler, A. Optical properties of coated black carbon aggregates: numerical simulations, radiative forcing estimates, and size-resolved parameterization scheme. *Atmos. Chem. Phys.,* ***2021****, 21, 12989–13010, https://doi.org/10.5194/acp-21-12989-2021.*

Sheridan P.J., Ogren J.A. Observations of the vertical and regional variability of aerosol optical properties over central and eastern north america. *J Geophys Res D Atmos.* ***1999****, 104(D14):16793-16805; DOI 10.1029/1999JD900241.*

Sorensen, C. M. Light scattering by fractal aggregates: A review. *Aerosol Sci. Technol.,* ***2001****, 35, 648–687,* https://doi.org/10.1080/02786820117868.Virkkula, A. Modeled source apportionment of black carbon particles coated with a light-scattering shell, *Atmos. Meas. Tech.,* ***2021****, 14, 3707–3719; DOI https://doi.org/10.5194/amt-14-3707-2021.*

Voshchinnikov N.V., Mathis J.S. Calculating cross sections of composite interstellar grains. *Astrophys J.* ***1999****, 526(1 PART 1):257-264; DOI 10.1086/307997.*

Wu Z.S., Wang Y.P. Electromagnetic scattering for multilayered sphere: Recursive algorithms. *Radio Sci.* ***1991****, 26(6):1393-1401; DOI 10.1029/91RS01192.*

Zhou Z., Xie M., Wang Z., Qi F. Determination of absolute photoionization cross-sections of aromatics and aromatic derivatives. *Rapid Commun Mass Spectrom.* ***2009****, 23(24):3994-4002; DOI 10.1002/rcm.4339.*

Zhou Z., Xie M., Wang Z., Qi F. Determination of absolute photoionization cross-sections of aromatics and aromatic derivatives. *Rapid Commun Mass Spectrom.* ***2009****, 23(24):3994-4002; DOI 10.1002/rcm.4339.*
